# Evaluation of Probiotic Properties and Antimicrobial Substances Produced by Five Lactic Acid Bacteria Against Foodborne and Spoilage Pathogens

**DOI:** 10.1002/mbo3.70052

**Published:** 2026-09-25

**Authors:** Md. Moklesur Rahman, Awis Qurni Sazili, Siti Aqlima Ahmad, Khalilah Abdul Khalil, Mohammad Rashedi Ismail‐Fitry, Md. Sazedul Karim Sarker

**Affiliations:** ^1^ Halal Products Research Institute Universiti Putra Malaysia UPM Serdang Selangor Malaysia; ^2^ Poultry Research Center Bangladesh Livestock Research Institute (BLRI) Savar Dhaka Bangladesh; ^3^ Faculty of Agriculture Universiti Putra Malaysia UPM Serdang Selangor Malaysia; ^4^ Faculty of Biotechnology and Bimolecular Science Universiti Putra Malaysia UPM Serdang Selangor Malaysia; ^5^ Faculty of Applied Sciences Universiti Teknologi MARA Shah Alam Selangor Malaysia; ^6^ Faculty of Food Science and Technology Universiti Putra Malaysia UPM Serdang Selangor Malaysia

**Keywords:** antimicrobial substance, foodborne pathogens, lactic acid bacteria, postbiotics, spoilage bacteria

## Abstract

The rise of multidrug‐resistant (MDR) pathogens presents a major challenge to food safety, particularly in preservation approaches. Lactic acid bacteria (LAB) gain probiotic recognition by effectively controlling MDR pathogens using antimicrobial substances (AMSs) and bacteriocin‐like inhibitory substances (BLISs). The study evaluated the probiotic properties and AMS production potential of five LAB strains: *Lactiplantibacillus plantarum* NBRC 3070 (LP), *Lactobacillus acidophilus* ATCC 4356 (LA), *Lacticaseibacillus casei* ATCC 393 (LC), *Lacticaseibacillus rhamnosus* GG ATCC 53103 (LGG), and *Bifidobacterium animalis* subsp. *lactis* ATCC 27673 (BAL). AMS was extracted from neutralized and concentrated cell‐free supernatant and tested for antimicrobial activities against common foodborne pathogens using minimum inhibitory concentrations (MICs). All LAB strains showed antagonistic activity, with LP demonstrating the most substantial antagonism. Four strains are resistant to multiple antibiotics but remain susceptible to azithromycin and tetracycline, while LP is resistant to all tested antibiotics. LP and LC demonstrated the highest probiotic potential based on cell surface hydrophobicity and aggregation abilities. Optimal AMS activity (85.36%–91.29%) occurred between 24 and 36 h at 37°C or 28–48 h at 30°C. LGG exhibited the highest AMS activity with the lowest MIC against *Salmonella enterica serovar* Typhimurium. Enzyme sensitivity confirmed the peptide nature of BLIS produced by BLIS from LP, LA, LC, and LGG. In conclusion, LAB strains demonstrated significant probiotic traits and the ability to produce AMS, especially BLIS, from four strains, with LP and LC showing the most potential. These findings support their application as natural biopreservation against MDR pathogens; however, further validation in the food system is required.

## Graphical abstract

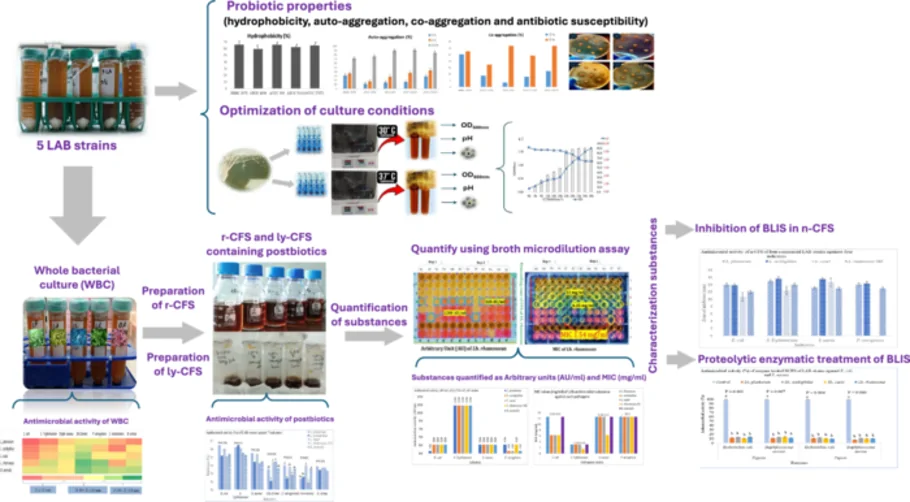

**Abbreviations**
AMR: antimicrobial resistance
AMSs: antimicrobial substances
ATCC: American type culture collection
BLISs: bacteriocin‐like inhibitory substances
BSA: bovine serum albumin
CFS: cell‐free supernatant
CFU: colony forming unite
EFSA: European Food Safety Authority
EU: European Union
FAO: Food and Agriculture Organization
FDA: the United States Food and Drug Administration
H_2_O_2_
: hydrogen peroxide
HCL: hydrochloric acid
LAB: lactic acid bacteria
LB: Luria Bertani
ly‐CFS: lyophilized cell‐free supernatant
MDR: multidrug resistance
MIC: minimum inhibitory concentration
MRS: de Man Rogosa Sharpe agar
NaOH: sodium hydroxide
NBRC: National Institute of Technology and Evaluation, Biological Resource Center
NITE: National Institute of Technology and Evaluation
NRRL: USDA‐ARS Culture Collection
n‐CFS: neutralized cell‐free supernatant
OD: optical density
r‐CFS: untreated cell‐free supernatant
SD: standard deviation
SEM: standard error of mean
UiTM: Universiti Teknologi MARA
WBC: whole bacterial culture
WHO: World Health Organization

## 1 Introduction

The rapid emergence of antimicrobial resistance (AMR) poses a growing global threat, particularly within the food industry, where it hampers and complicates the control of multidrug‐resistant (MDR) foodborne pathogens and spoilage organisms (Samreen et al. 2021). Pathogen resistance to antibiotics and chemical preservatives complicates foodborne disease treatments, impacting public health, food safety, product quality, and consumer trust (Lianou et al. 2017; Zhaxybayeva et al. 2020). Among the most frequently implicated pathogens in food contamination, like, *Escherichia coli*, *Staphylococcus aureus, Salmonella* spp., *Shigella* spp., *Pseudomonas aeruginosa*, *Serratia marcescens*, and *Bacillus cereus*, are commonly associated with contamination in meat, dairy, water, and ready‐to‐eat foods, contributing to both illness and food waste (Amenu and Bacha 2024; Eji et al. 2023; Thuy et al. 2024). While conventional methods such as synthetic chemicals and heat treatment have been widely used, they often leave toxic residues and are less effective against MDR pathogens (Afrin et al. 2021). These limitations have increased demand for safer, ecofriendly, and natural alternatives for consumers and industries (Teshome et al. 2022). In response to these challenges, attention has shifted toward biopreservation strategies involving probiotic‐derived compounds, including antimicrobial substances (AMSs). They have gained significant attention for their potential to enhance food safety and the shelf life of perishable foods without compromising nutritional and sensory quality.

Lactic acid bacteria (LAB) are among the most widely studied probiotic microorganisms due to their long history of safe use in food fermentation and are generally recognized as safe. They are known for their ability to regulate microbial communities in food and the guts through the secretion of various antimicrobial metabolites. These include organic acids (lactic acid and acetic acid), hydrogen peroxide (H_2_O_2_), bacteriocins, and bacteriocin‐like inhibitory substances (BLISs) (Abdul Hakim et al. 2023; Castellano et al. 2017; Cirat et al. 2024). Such compounds contribute to the inhibition of both Gram‐positive and Gram‐negative bacteria through a range of mechanisms, including disruption of membrane integrity, inhibition of essential enzymes, and interference with DNA or protein synthesis (Fernandes et al. 2021; Ibrahim et al. 2021). Among these antimicrobial agents, BLIS stands out due to its emerging relevance and broad‐spectrum antimicrobial activity. While similar to bacteriocins in function, BLIS is less well‐characterized and may possess unique narrow to broad‐spectrum activity and mode of action. These agents are associated with a lower risk of resistance in target pathogens compared with synthetic antibiotics. They can act as either bacteriostatic or bactericidal agents and provide broad‐spectrum antimicrobial activity without harming the producing LAB strain (Leite et al. 2016; Moradi et al. 2021). BLIS exerts antimicrobial effects by forming pores in the membranes of target bacteria, causing leakage of ions, dissipation of membrane potential, and eventual cell death. In some cases, they may also interfere with cellular biosynthesis pathways, further enhancing their efficacy (Fernandes et al. 2021).

To ensure the safe and effective application of these LAB strains in food systems, their probiotic potential must be comprehensively characterized. International regulatory bodies, such as the Food and Agriculture Organization, World Health Organization, and European Food Safety Authority (EFSA), emphasize the importance of thorough probiotic characterization. Key criteria include proven antagonistic activity against toxigenic microorganisms, favorable cell surface properties (such as hydrophobicity, autoaggregation, and coaggregation), the capacity to inhibit pathogen adhesion, and the absence of transferable AMR genes (Delgado et al. 2020; Denkova‐Kostova et al. 2023; W. Ma et al. 2024). Importantly, the antimicrobial efficacy of LAB metabolites is significantly influenced by environmental parameters like incubation temperature, pH, and fermentation time, which must be optimized to maximize AMS and BLIS (Md Sidek et al. 2018; Elazzazy et al. 2024).

Although these aspects are well recognized, many existing studies remain limited in scope. Despite the large volume of literature on LAB, many previous studies have focused on individual strains or isolated probiotic traits without offering a comprehensive, comparative analysis under standard and optimized conditions. Moreover, while AMS production by LAB has been widely reported, limited studies have characterized the biochemical nature of BLIS or examined the environmental factors influencing their yield and activity. To address these gaps, the current study selected five commercially relevant LAB strains with established probiotic backgrounds for a more detailed investigation. In particular, LAB strains such as *Lactiplantibacillus plantarum* NBRC 3070, *Lactobacillus acidophilus* ATCC 4356, *Lacticaseibacillus casei* ATCC 393, *Lacticaseibacillus rhamnosus* GG ATCC 53103, and *Bifidobacterium animalis* subsp. *lactis* ATCC 27673 were selected in this study due to its relevance as starter cultures in food fermentation and established probiotic potential. However, other strains of *L. plantarum*, *L. acidophilus*, *L. casei*, *L. rhamnosus GG, and B. animalis* subsp. *lactis* have been extensively studied (D'agostino et al. 2024; Dardmeh et al. 2023; Derebasi et al. 2024; Echegaray et al. 2023; Y. S. Lee et al. 2023; Roldán‐Pérez et al. 2023), while selected strains of NBRC and ATCC remain underexplored in terms of their comprehensive probiotic attributes and AMS production. Furthermore, little is known about the interplay between surface adhesion traits and AMS production in these particular strains. Previous studies have not thoroughly investigated the optimal conditions for producing the maximum AMS production and peptide nature and characterization of BLIS in these specific strains. Likewise, the cell surface properties such as hydrophobicity and aggregation ability, critical for adhesion, colonization, and microbial inhibition, are seldom considered holistically alongside BLIS activity. Most prior studies have focused on individual aspects of probiotic attributes and AMS production; however, integrated assessment remains limited.

Therefore, the present study aims to fill these knowledge gaps by evaluating the probiotic properties, antimicrobial efficacy, antibiotic susceptibility, and physicochemical properties of AMS produced by five selected LAB strains. Specific objectives include (i) assessing cell surface properties (hydrophobicity, autoaggregation, and coaggregation), (ii) optimizing growth conditions for maximizing AMS production, (iii) quantifying AMS activity in arbitrary unit (AU) and minimum inhibitory concentration (MIC), and (iv) characterizing the proteinaceous nature of BLIS. This study hypothesized that five selected LAB strains would exhibit distinct probiotic characteristics and produce AMS under standardized conditions with effective inhibitory activity against both Gram‐positive and Gram‐negative MDR pathogens, including measurable enzyme sensitivity. Notably, this study will uniquely investigate the establishment of a direct correlation between AUs and MICs, a relationship that is seldom quantified. The goal of this study is to determine the feasibility of using these strains and their metabolites as safe, natural biopreservatives for combating AMR and MDR foodborne pathogens and improving food safety.

## 2 Materials and Methods

### 2.1 Test Strains, Media, and Culture Conditions

Five probiotic LAB strains were used: *L. plantarum* NBRC 3070, *L. acidophilus* ATCC 4356, *L. casei* ATCC 393, *L. rhamnosus* GG ATCC 53103, and *B. animalis* subsp. *lactis* ATCC 27673. These strains were selected based on their probiotic applications, documented abilities to produce antimicrobial compounds, and their relevance in previous studies for controlling pathogens. Moreover, they are widely used in food and probiotic formulations and are commercially available, which enhances their translational potential for industry applications. To evaluate their antimicrobial potential, both whole bacterial cultures (WBCs) and cell‐free supernatant (CFS) were tested against seven foodborne and spoilage indicator bacteria, including five Gram (*E. coli* ATCC 25922, *Salmonella enterica serovar* Typhimurium ATCC 14028, *Shigella sonnei* ATCC 25931, *P. aeruginosa* ATCC 10145, *S. marcescens* ATCC 14756) and two Gram‐positive (*S. aureus* ATCC 43300 and *B. cereus* ATCC 14579) bacteria (Table S1). All LAB and indicator strains were kindly provided by the Faculty of Applied Science, School of Biology, Universiti Teknologi MARA, Malaysia. LAB strains were cultured in de Man, Rogosa, and Sharpe (MRS) broth (Condalab, Spain) under anaerobic conditions (RS Biotech, UK) at 37°C for 24 h. Indicator strains were cultured in Luria Bertani (LB) broth (Condalab, Spain) under aerobic conditions at 28°C–37°C for 24 h. Subcultures were prepared by inoculating 1% (v/v) into fresh broth for three consecutive transfers. Final cultures were incubated at 37°C for 48 h and used as experimental inocula. All strains were stored at −80°C (ULTRA‐LOW temperature Freezer, SANYO, Japan) in 40% (v/v) glycerol (Sigma‐Aldrich, EU) for long‐term preservation.

### 2.2 Evaluation of Probiotic Properties

#### 2.2.1 Antimicrobial Spectrum of Whole Bacterial Cells of LAB Strains

The antimicrobial activity of WBC from LAB strains was assessed using the agar well diffusion method, following the procedure of Ohaegbu et al. (2022), with minor modifications. Each LAB strain was grown in MRS broth at 37°C for 24 h until reaching an optical density (OD_600nm_) of 0.42–0.47, measured using a UV–Vis Spectrophotometer (Multiskan GO, Thermo Scientific, Finland). Indicator strains were cultured in LB broth and adjusted to OD_600nm_ values of 0.24–0.28. Sterile LAB agar plates were swabbed uniformly with the bacterial cell suspensions. After allowing the plates to dry for 1 h at room temperature, wells (6 mm diameter) were punched into the agar using the sterile pipette tips. Each well was filled with 100 µL of the active LAB culture and allowed to diffuse for 30 min at room temperature. Plates were incubated for 24 h at 37°C. MRS broth and 0.02% acetic acid were used as negative and positive controls, respectively. After incubation, zones of inhibition were measured in millimeters. Antimicrobial activity was interpreted as follows: < 8 mm (not sensitive), 9–14 mm (sensitive), 15–19 mm (very sensitive), and > 20 mm (extremely sensitive) (Moradi et al. 2020). All assays were performed at least three times.

#### 2.2.2 Preparation of Raw, Neutralized, and Lyophilized CFS (ly‐CFS)

Following confirmation of antimicrobial activity in WBC, the AMS produced by LAB strains were further evaluated in their respective CFS. Each LAB strain (2%, v/v inoculum) was suspended in 50 mL MRS broth and incubated anaerobically at 37°C for 48 h as described by Ye et al. (2021). After incubation, cultures were centrifuged at 10,000 rpm for 20 min at 4°C (Eppendorf Centrifuge 5430 R, Germany). The resulting supernatants were collected and their pH measured using a calibrated pH meter (Mettler Toledo SevenCompact S220, Schwiez). The CFS was divided into two equal portions: one portion was filter‐sterilized through a 0.22‐μm PVDF Syringe filter (Merck, Germany) and stored at −20°C for subsequent use, considered as untreated CFS (r‐CFS). The second portion was adjusted to pH 6.0– 6.5 with 5 M NaOH (Sigma, Germany) to eliminate the effects of organic acids and hydrogen peroxide, followed by heat treatment at 80°C for 10 min (Amin et al. 2020; Ren et al. 2022). This preparation was used for evaluating BLIS and termed as neutralized CFS (n‐CFS). The pH of both r‐CFS and n‐CFS was calculated immediately after preparation (Table S2). For lyophilization, n‐CFS were frozen at −40°C, subjected to 100 mTorr pressure, and dried at −60°C. The resulting ly‐CFS was stored and used in subsequent experiments (Hossain et al. 2021).

#### 2.2.3 Antimicrobial Spectrum of the CFS of LAB Strains

To evaluate the antimicrobial spectrum of AMS produced in their r‐CFS of LAB strains, two complementary assays were employed: the agar well diffusion assay (as described in Section 2.2.1) and the broth microdilution assay using 96‐well plates. For the broth microdilution assay, 40 µL of r‐CFS (20% v/v) was added to each well of sterile 96‐well cell culture plates (SPL, Korea). Each well was then inoculated with 10 μL (5%, v/v) of overnight‐activated indicator strain, adjusted to an OD_600nm_ of 0.28–0.30. The final volume in each well was brought to 200 μL using LB broth. Indicator strain cultured in LB broth without r‐CFS was used as a growth control, whereas MRS broth (pH 6.8) diluted in LB broth was used as a negative control. After 24 h incubation at 37°C, bacterial growth was monitored by measuring OD_600nm_ using a spectrophotometer. The percentage (%) of growth inhibition was calculated according to Equation (1):

(1) $$\text{Inhibition}\;\left(\%\right)=\left\{\left({\mathrm{OD}}_{600\text{nm}}\;\text{control}-{\mathrm{OD}}_{600\text{nm}}\;\text{sample}\right)/{\mathrm{OD}}_{600\text{nm}}\;\text{control}\right\}\times 100,$$

where

OD_600nm_ control = absorbance of the growth control (no r‐CFS),

OD_600nm_ sample = absorbance of the test sample (with r‐CFS).

All tests were conducted in triplicate (*n* = 3). Results were expressed as mean ± standard deviation and used to determine the antimicrobial spectrum of the LAB‐derived r‐CFS against seven indicator strains.

#### 2.2.4 Antibiotic Susceptibility Analysis of LAB Strains

The antibiotic sensitivity of LAB strains was evaluated using the disc diffusion method on Mueller–Hinton (MH) agar (Condalab, USA), following Clinical and Laboratory Standards Institute (CLSI) guidelines (CLSI 2023) with minor modifications adapted from (Humphries et al. 2021). LAB strains were cultured in MRS broth and incubated at 37°C for 24 h. The bacterial suspensions were adjusted to appropriate turbidity and swabbed evenly onto MH agar plates using sterile cotton swabs. After drying for 15 min at room temperature, commercial antibiotic discs (Oxide, Thermo Scientific) were placed on the agar surface. The antibiotics tested included: azithromycin (30 µg), ciprofloxacin (5 µg), gentamycin (10 µg), norfloxacin (10 µg), penicillin (10 µg), tetracycline (30 µg), and streptomycin (10 µg) (Table S3). Plates were incubated at 37°C for 24 h. The diameters of the inhibition zones (including disc) were measured in millimeters. Results were interpreted as sensitive (S), intermediate (I), or resistant (R) according to CLSI breakpoints. All assays were performed in triplicate, and results were expressed as mean ± standard deviation.

#### 2.2.5 Cell Surface Hydrophobicity of LAB Strains

The surface hydrophobicity of five LAB strains was evaluated using the microbial adhesion to hydrocarbon method as described by Salas‐Tovar et al. (2021), with xylene as the nonpolar solvent. Briefly, LAB cultures were grown in MRS broth (1%, v/v) and incubated at 37°C for 24 h. The cells were harvested by centrifugation (8000 rpm, 10 min, 4°C), washed twice with phosphate‐buffered saline (PBS, 1.54 mM KH_2_PO_4_, 0.1 mM NaCl, 2.71 mM Na_2_HPO_4_·7H_2_O, pH 7.4), and resuspended in the same buffer (G‐Bioscience, USA) to an OD_600nm_ of 0.50–0.55 (≈ 10^8^ CFU/mL). A 3.0‐mL cell suspension was mixed with 1.0 mL of xylene and vortexed gently. After standing at room temperature for 10 min, the initial absorbance (*A*_0_) was measured at 600 nm. Following a 30‐min incubation to allow phase separation, the aqueous phase absorbance (*A_t_*) was measured. Hydrophobicity (%) was calculated following equation (2):

(2) $$\mathrm{Hydrophobicity}\;\left(\%\right)=\left[\frac{A_{0}-A_{t}}{A_{0}}\right]\times 100.$$

#### 2.2.6 Autoaggregation Assay of LAB Strains

Autoaggregation assay was performed according to B. Wang et al. (2024) with minor modifications. LAB strains were freshly grown in MRS broth at 37°C for 24 h, harvested by centrifugation (8000 rpm, 10 min, 4°C), collected, and rinsed twice with PBS. The cells were resuspended in PBS to an OD₆₀₀ of 0.62–0.68 (≈ 10^8^ CFU/mL). A 2.0‐mL suspension was vortexed for 10 s and incubated at room temperature for 5 h. Absorbance was measured at 600 nm at 0 h (*A*_0_) and 2, 5, and 24 h (*A_t_*). The percentage of autoaggregation (Auto‐A%) was calculated using the following equation (3):

(3) $$\mathrm{Auto}‐A\%=\left[\frac{A_{0}-A_{t}}{A_{0}}\right]\times 100.$$

#### 2.2.7 Coaggregation Assay of LAB Strains

The coaggregation ability of LAB strains with two indicator pathogens, *E. coli* ATCC 25922 and *S. aureus* ATCC 43300 was determined as per B. Wang et al. (2024). The LAB and indicator strains were grown and prepared as described above. Equal volumes (2 mL) of LAB and pathogen suspension were mixed, vortexed for 10 s, and incubated for 2 and 5 h. Absorbance at 600 nm was measured for each strain alone (*A_x_*, *A_y_*) and for the mixed suspension (*A*_*x*+*y*_). The coaggregation percentage (Co‐A%) was calculated using Equation (4):

(4) $$\mathrm{Co}‐A\%=\left[1-\frac{A_{\left(x+y\right)}}{{(A}_{x}+A_{y})/2}\right]\times 100.$$

All experiments were performed in triplicate, and results were reported as mean ± standard deviation.

### 2.3 Production Potential of AMSs From LAB Strains

To evaluate the growth and cumulative production of AMS, five LAB strains were cultured in MRS broth. Each strain (1%, v/v, 500 µL) was inoculated into 50 mL of MRS broth and incubated anaerobically at 30°C and 37°C for 48 h. Bacterial growth (OD_600nm_), pH value, and AMS activity in CFS were monitored at 4 h intervals, following J. Ma et al. (2020). The OD_600nm_ and pH values were tested using a spectrophotometer and pH meter (Mettler Toledo SevenCompact S220, Switzerland), respectively. The antibacterial activity of CFS was assessed via the broth microdilution assay described in Section 2.2.2. *S. aureus* ATCC 43300 was selected as an indicator due to its broad sensitivity spectrum. Inhibition percentage (*I* %) was calculated as described previously.

### 2.4 Qualification of Antimicrobial Activity

#### 2.4.1 AU/mL Determination

The antimicrobial activity of AMS in ly‐CFS was quantified using a broth microdilution assay, adapted from (Ansari et al. 2021). Indicator strains included *E. coli* ATCC 25922, *S. Typhimurium* ATCC 14028, *S. aureus* ATCC 43300, *P. aeruginosa* ATCC 10145, *S. marcescens* ATCC 14756, and *B. cereus* ATCC 14579. In each well of a 96‐well plate, 50 µL of a twofold serially diluted ly‐CFS (starting concentration, 100 mg/mL in MRS medium) was mixed with 10 µL of indicator strain (OD_600nm_ = 0.11–0.15), and the final volume was adjusted to 100 μL using 50 μL of LB broth. Cultures of indicator strains without ly‐CFS were used as a positive control, whereas MRS medium in LB broth was used as a negative control. After 16 h incubation at 37°C, bacterial growth inhibition (%) was determined spectrophotometrically at OD_600nm_. One AU/mL was defined as the reciprocal of the highest dilution that inhibited at least 50% bacterial growth compared with the positive control. All tests were performed in triplicate.

#### 2.4.2 MICs Determination

MICs of AMS from ly‐CFS were determined using a twofold serial dilution method, followed by Bajpai et al. (2016). In a 96‐well plate, 50 µL of enriched LB broth was added to each well. Ly‐CFS (stock: 100 mg/mL) was serially diluted in LB broth to final concentrations of 100, 50, 25, 12.5, 6.25, 3.12, and 1.56 mg/mL. Then, 50 μL of standardized indicator inoculum (OD_600nm_ = 0.10–0.14) was incorporated into each well. Indicator‐only wells served as positive controls, and MRS in LB broth served as negative controls. Plates were incubated at 37°C for 24 h. MIC was defined as the lowest concentration of ly‐CFS that resulted in ≥ 90% inhibition of bacterial growth relative to the positive control (G. Wang and Zeng 2022; Wayah and Philip 2018). All experiments were performed in triplicate (*n* = 3).

### 2.5 Identification and Characterization of BLISs

To assess BLIS activity, ly‐CFS was evaluated using the agar well diffusion assay against seven indicator strains, as described earlier. To confirm the proteinaceous nature of the BLIS, ly‐CFS was treated with proteolytic enzymes: pepsin (pH 3.0, 37°C, 0.1 M HCl), trypsin (pH 7.5, 37°C, 10 mM PBS), and papain (pH 7.5, 37°C, 10 mM PBS), each at a final concentration of 1.0 mg/mL (H. Du et al. 2018; Peng et al. 2023). Enzymatic treatments were performed at 37°C for 4 h under sterilized conditions and terminated by heating at 100°C for 5 h. The residual antibacterial efficacy of enzyme‐treated samples was measured using a broth microdilution assay against *E. coli* ATCC 25922, *S. Typhimurium* ATCC 14028, *S. aureus* ATCC 43300, and *P. aeruginosa* ATCC 10145. Controls included r‐CFS (positive control) prepared with MRS broth with enzymes at specified concentrations and CFS at the original pH without heat or enzyme treatments (negative control). All tests were conducted in triplicate, and results were reported as mean values.

### 2.6 Statistical Analysis

The experiments followed a completely randomized design, with LAB strains and indicator pathogens considered as independent variables. Statistical analysis was conducted using SPSS version 25 (IBM, USA, 2023). One‐way analysis of variance (ANOVA) was used to evaluate significant differences among treatment means. For time‐dependent data, repeated‐measure ANOVA was performed. When ANOVA revealed significant differences (*p* < 0.05), Duncan's Multiple Range Test was used as a post hoc test to compare group means. The difference in antibacterial activity among LAB strains and overtime was performed using univariate analysis of General Linear Model (GLM) with univariate analysis. Each experiment was replicated at least three times (*n* = 3), and the data results were stated as the mean value ± standard deviation.

## 3 Results

### 3.1 Evaluation of Probiotic Properties

#### 3.1.1 Antibacterial Spectrum of WBCs

**Figure 1:**
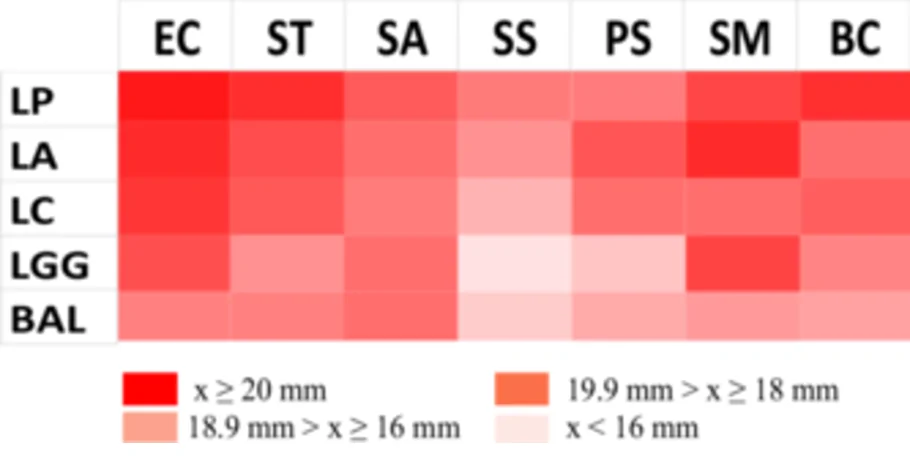
Inhibition zones (heatmap) including five tested LAB strains against seven indicator strains. Explanation: EC, *Escherichia coli* ATCC 25922; ST, *Salmonella enterica serovar* Typhimurium ATCC 14028; SA, *Staphylococcus aureus* ATCC 43300; SS, *Shigella sonnei* ATCC 25931; PA, *Pseudomonas aeruginosa* ATCC 10145; SM, *Serratia marcescens* ATCC 14756; BC, *Bacillus cereus* ATCC 14579; LP, *Lactiplantibacillus plantarum* ATCC 3070; LA, *Lactobacillus acidophilus* ATCC 4356; LC, *Lacticaseibacillus casei* ATCC 393; LGG, *Lacticaseibacillus rhamnosus GG* ATCC 53103; BAL, *Bifidobacterium animalis* subsp. *lactis* ATCC 27673; *x*, antimicrobial activity. LAB, lactic acid bacteria.

The presence of antimicrobial activity in LAB strains was screened in this study using WBC against indicators, as shown in Figure 1. The heatmap shows that the LAB strains demonstrated antagonistic activity toward the seven indicators. The indicators were classified as “very sensitive” (15–22 mm) and “extremely sensitive” (> 20 or > 22 mm) based on the inhibition zones formed by the WBC, which ranged from 15.17 ± 1.32 to 23.05 ± 0.95 mm. Statistically significant differences (*p* < 0.01) were observed in antagonistic activity among the WBC of LAB strains, and sensitivity among the seven indicator strains, except *S. aureus* ATCC 43300 (Table S4). Among the LAB strains, *L. plantarum* NBRC 3070 produced the largest inhibition zone (21.44 ± 0.95 mm) against the indicators. Among the tested pathogens, *E. coli* ATCC 25922 showed the highest mean inhibition zone (21.61 ± 1.02 mm) when exposed to the WBC of all LAB strains (*p* < 0.01), followed by *S. marcescens* ATCC 14756 (20.52 ± 1.10 mm) and *S. Typhimurium* ATCC 14028 (20.22 ± 1.05 mm). In contrast, *S. sonnei* ATCC 25931 showed the lowest sensitivity (17.14 ± 0.80 mm).

#### 3.1.2 Antibacterial Spectrum of Postbiotics

**Figure 2:**
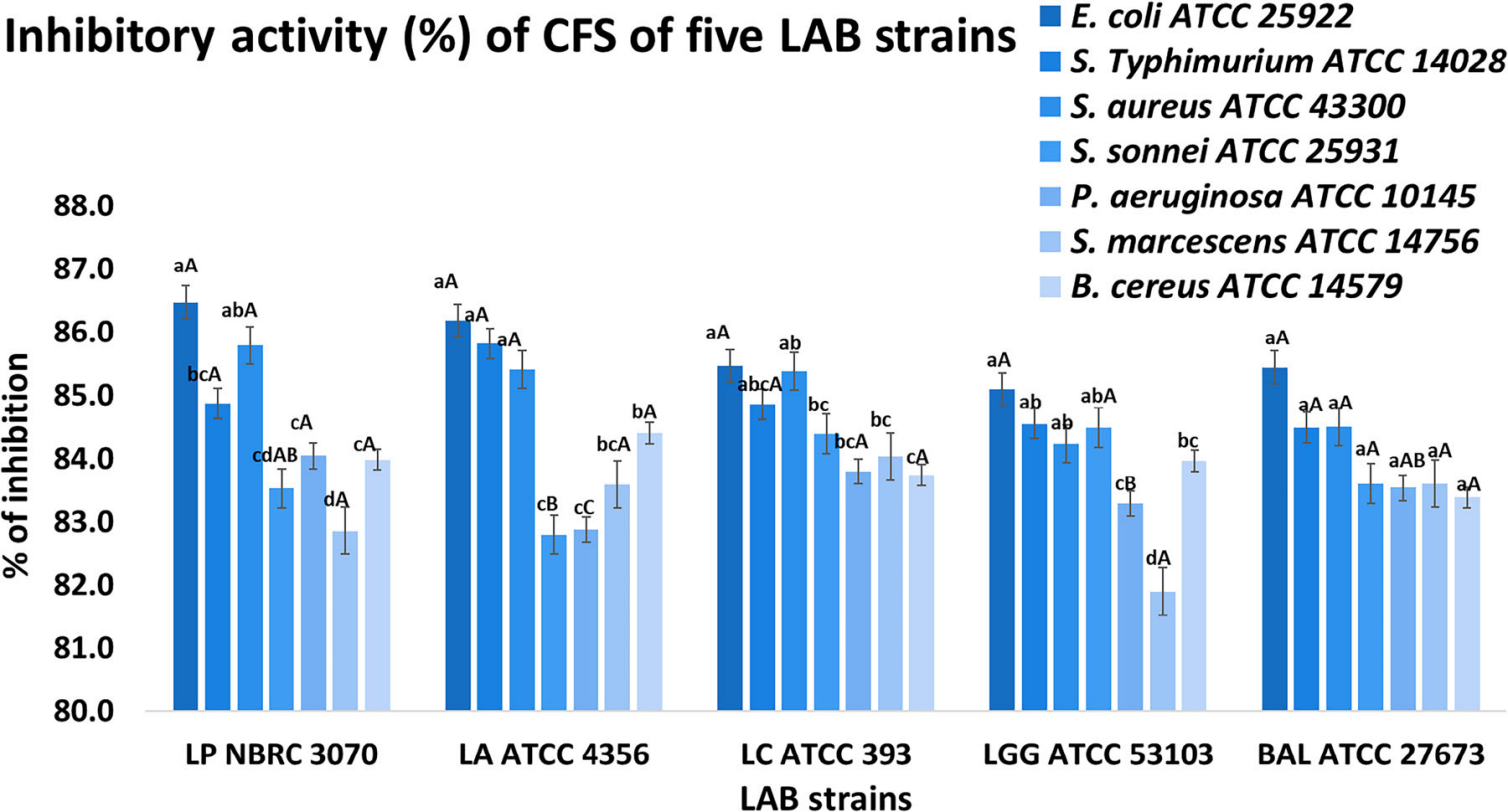
Inhibitory activity (%) of untreated cell‐free supernatant (r‐CFS) containing antimicrobial substances (AMSs) obtained from five LAB strains against seven indicator microorganisms determined by broth microdilution techniques. The error bars indicate the standard error of the mean (*n* = 3). Lowercase letters (a, b, c, and d) represent statistically significant differences among different indicator strains within each LAB strain. Uppercase letters (A, B, and C) represent statistically significant differences among different LAB strains within each indicator strain (*n* = 3, *p* < 0.05, Duncan's test). LP, r‐CFS obtained from *Lactiplantibacillus plantarum* NBRC 3070; LA, r‐CFS obtained from *Lactobacillus acidophilus* ATCC 4356; LC, r‐CFS obtained from *Lacticaseibacillus casei* ATCC 393; LGG, r‐CFS obtained from *Lacticaseibacillus rhamnosus* GG ATCC 53103, and BAL, r‐CFS obtained from *Bifidobacterium animalis* subsp. lactis ATCC 27673. LAB, lactic acid bacteria.

The antibacterial activity of r‐CFS of LAB strains containing AMS against the tested indicators was confirmed by the presence of inhibition zones on the agar well plates (mm) and growth inhibition activity (%) in a 96‐well cell culture plate, as presented in Table 1 and Figure 2, respectively. The table showed that the AMS consistently exhibited antibacterial activity against seven indicators, with the inhibition zones ranging from 14.75 to 23.67 mm. Among the LAB strains, *L. plantarum* NBRC 3070 (20.81 ± 1.08 mm), *L. acidophilus* ATCC 4356 (20.38 ± 1.12 mm), *L. casei* ATCC 393 (19.18 ± 0.98 mm), and *L. rhamnosus* GG ATCC 53103 (19.12 ± 0.98 mm) produced significantly larger inhibition zones (*p* < 0.01) compared with *B. animalis* subsp. *lactis* ATCC 27673, based on GLM analysis followed by Duncan's post hoc test. Among all indicators, *E. coli* ATCC 25922 exhibited the largest inhibition zones (range 22.75–23.67), followed by *S. marcescens* ATCC 14756 (range 21.17–22.75) across all AMS of LAB. Additionally, *S. aureus* ATCC 43300 (22.67 mm), *P. aeruginosa* ATCC 10145 (19.42 mm), and *S. sonnei* ATCC 25931 (17.75 ± 0.68 mm) were the most inhibited by the r‐CFS of *L. acidophilus* ATCC 4356, while the AMS of *L. plantarum* NBRC 3070 displayed the highest inhibition against *S. Typhimurium* ATCC 14028 (22.58 ± 1.04 mm), *P. aeruginosa* ATCC 10145 (19.42 mm ± 0.78 mm), and *B. cereus* ATCC 14579 (19.50 mm). In contrast, *S. aureus* ATCC 43300 was more strongly inhibited by the r‐CFS of *L. rhmanosus* GG ATCC 53103 (20.67 mm ± 1.18 mm). The inhibition produced by *B. animalis* ATCC 27673 was significantly lower (*p* < 0.05) than that of other *Lactobacillus* strains. Among all pathogens, *S. sonnei* ATCC 25931 was the least sensitive, with inhibition zones ranging from 15 to 18 mm across all LAB strains.

**Table 1:** Diameter (mm) of inhibition zones with well diffusion assay generated by untreated CFS (r‐CFS) of studied LAB strains against selected indicator strains for 24 h of incubation.

Pathogenic bacteria	LAB strains					SEM	*p* value
	*Lactiplantibacillus plantarum*	*Lactobacillus acidophilus*	*Lacticaseibacillus casei*	*Lacticaseibacillus rhamnosus* GG	*Bifidobacterium animalis* subsp. *lactis*		
*Escherichia coli*	23.67^aA^	22.75^aA^	23.00^aA^	22.83^aA^	19.98^bA^	0.443	0.000
*Salmonella enterica serovar* Typhimurium	22.58^aB^	22.67^aA^	18.50^bC^	17.92^bcC^	16.75^cC^	0.195	0.001
*Staphylococcus aureus*	20.25^aC^	19.42^bB^	20.42^aB^	20.67^aBC^	14.75^cE^	0.557	0.000
*Shigella sonnei*	17.50^aF^	17.75^aC^	15.00^cD^	16.00^bcD^	15.50^bcDE^	0.279	0.001
*Pseudomonas aeruginosa*	19.42^aD^	17.50^bC^	18.58^aC^	17.75^bCD^	15.83^cCDE^	0.351	0.000
*Serratia marcescens*	22.75^aB^	22.50^aA^	20.09^cB^	21.17^bcB^	21.17^bcB^	0.268	0.008
*Bacillus cereus*	19.50^aE^	18.08^bC^	18.67^abC^	17.50^cCD^	16.17^dCD^	0.281	0.006
SEM	0.522	0.313	0.508	0.579	0.727		
*p* value	0.000	0.000	0.000	0.000	0.000		

*Note:* Lowercase letters (a, b, c, and d) represent statistically significant differences among different LAB strains within each indicator strain (*n* = 3, *p* < 0.05, Duncan's test). Uppercase letters (A, B, C, D, and E) represent statistically significant differences among different indicator strains within each LAB strain (*n* = 3, *p* < 0.05, Duncan's test).

Abbreviations: CFS, cell‐free supernatant; LAB, lactic acid bacteria; SEM, standard error of mean.

The inhibition percentages derived from the broth microdilution assay also varied significantly on the differences among the five LAB strains (*p* < 0.05) and indicator strains (*p* < 0.05) (Figure 2). *L. plantarum* NBRC 3070 and *L. casei* ATCC 393, exhibited the highest inhibition against *E. coli* ATCC 25922 (85.10%–86.47%), *S. Typhimurium* ATCC 14028 (86.52%–85.29%), and *S. aureus* ATCC 43300 (84.23%–85.49%), yet they were nonsignificant (*p* > 0.05). Notably, *L. casei* ATCC 393 and *L. rhamnosus* GG ATCC 53103 displayed a high inhibitory effect against *S. sonnei* ATCC 25931 (84.39% and 84.49%, respectively), while *L. casei* ATCC 393 also exhibited strong inhibition against *S. marcescens* ATCC 14756 (84.04%) compared with other LAB strains. Overall, AMS from LAB strains demonstrated the strongest activity against *E. coli* ATCC 25922 compared with other indicators.

#### 3.1.3 Antibiotic Susceptibility Analysis

**Figure 3:**
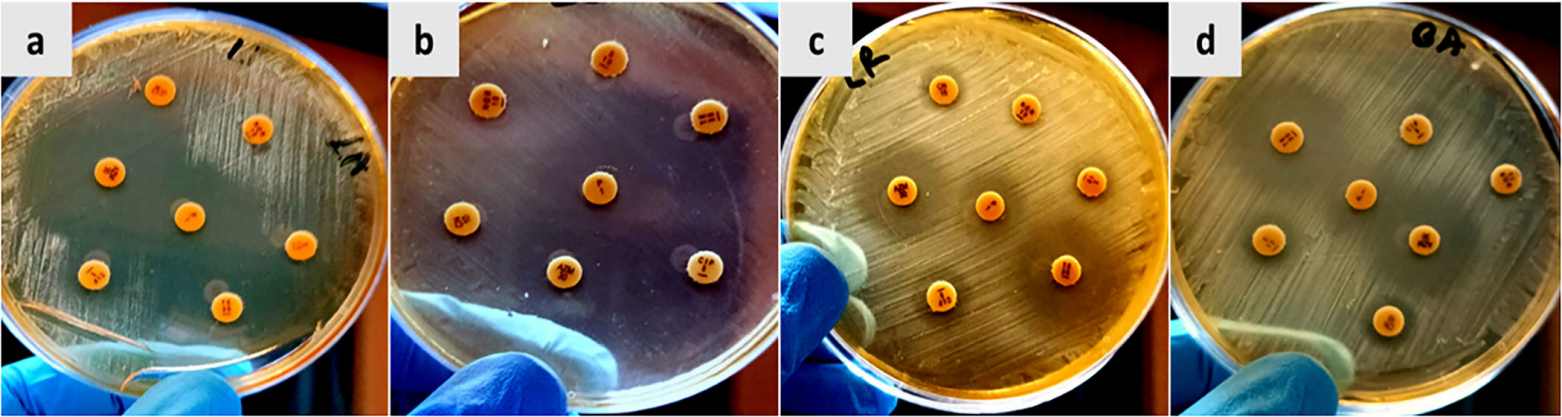
The disk diffusion assay determined the antibiotic susceptibility pattern of representative lactic acid bacteria strains against seven different antibiotics: AZM, Azithromycin; CIP, Ciprofloxacin; CN, Gentamycin; NOR, Norfloxacin; P, Penicillin; S, Streptomycin; TE, Tetracycline. (a) *Lactobacillus acidophilus* ATCC 4356, (b) *Lacticaseibacillus casei* ATCC 393, (c) *Lacticaseibacillus rhamnosus* GG ATCC 53103, and (d) *Bifidobacterium animalis* subsp. *lactis* ATCC 27673.

The antibiotic susceptibility profiles of the five LAB strains against seven antibiotics are presented in Table 2. Most LAB strains were susceptible to azithromycin, tetracycline, ciprofloxacin, and penicillin, with inhibition zones ranging from 21 to 39 mm. All strains except *L. plantarum* NBRC 3070 were sensitive to azithromycin (21–39 mm) and tetracycline (13–26 mm). Resistance to gentamycin, streptomycin, and norfloxacin was observed across all strains, with inhibition zones either absent or ≤ 13 mm. *L. plantarum* NBRC 3070 displayed no susceptibility to any tested antibiotics, with resistance zones of 12–13 mm. *B. animalis* subsp. *lactis* ATCC 27673 was resistant to ciprofloxacin (no measurable zone), while *L. casei* ATCC 393 was resistant to penicillin. Disk diffusion assay results of four representative strains are depicted in Figure 3.

**Table 2:** Antibiotic susceptibility pattern of LAB strains against different antibiotics recorded in terms of zone of inhibition (mm) by disc diffusion assay.

LAB strains	Strains ID	Antibiotics						
		AZM	CIP	CN	NOR	P	TE	S
*Lactiplantibacillus plantarum*	NBRC 3070	R	R	R	R	R^12^	R^13^	R
*Lactobacillus acidophilus*	ATCC 4356	S^39^	I^15^	R	R	S^29^	S^26^	R
*Lacticaseibacillus casei*	ATCC 393	S^21^	S^18^	R	R	R	I^17^	R
*Lacticaseibacillus rhamnosus* GG	ATCC 53103	S^24^	R^12^	R^11^	R	S^18^	S^30^	R
*Bifidobacterium animalis* subsp. *lactis*	ATCC 27673	S^25^	R	R^12^	R	S^19^	S^26^	R^11^

*Note:* Superscripts denote zones of inhibition (ZOI). I, intermediate susceptible; R, resistant; S, susceptible.

Abbreviations: AZM, Azithromycin; CIP, Ciprofloxacin; CN, Gentamycin; LAB, lactic acid bacteria; NOR, Norfloxacin; P, Penicillin; S, Streptomycin; TE, Tetracycline.

#### 3.1.4 Cell Surface Hydrophobicity

**Figure 4:**
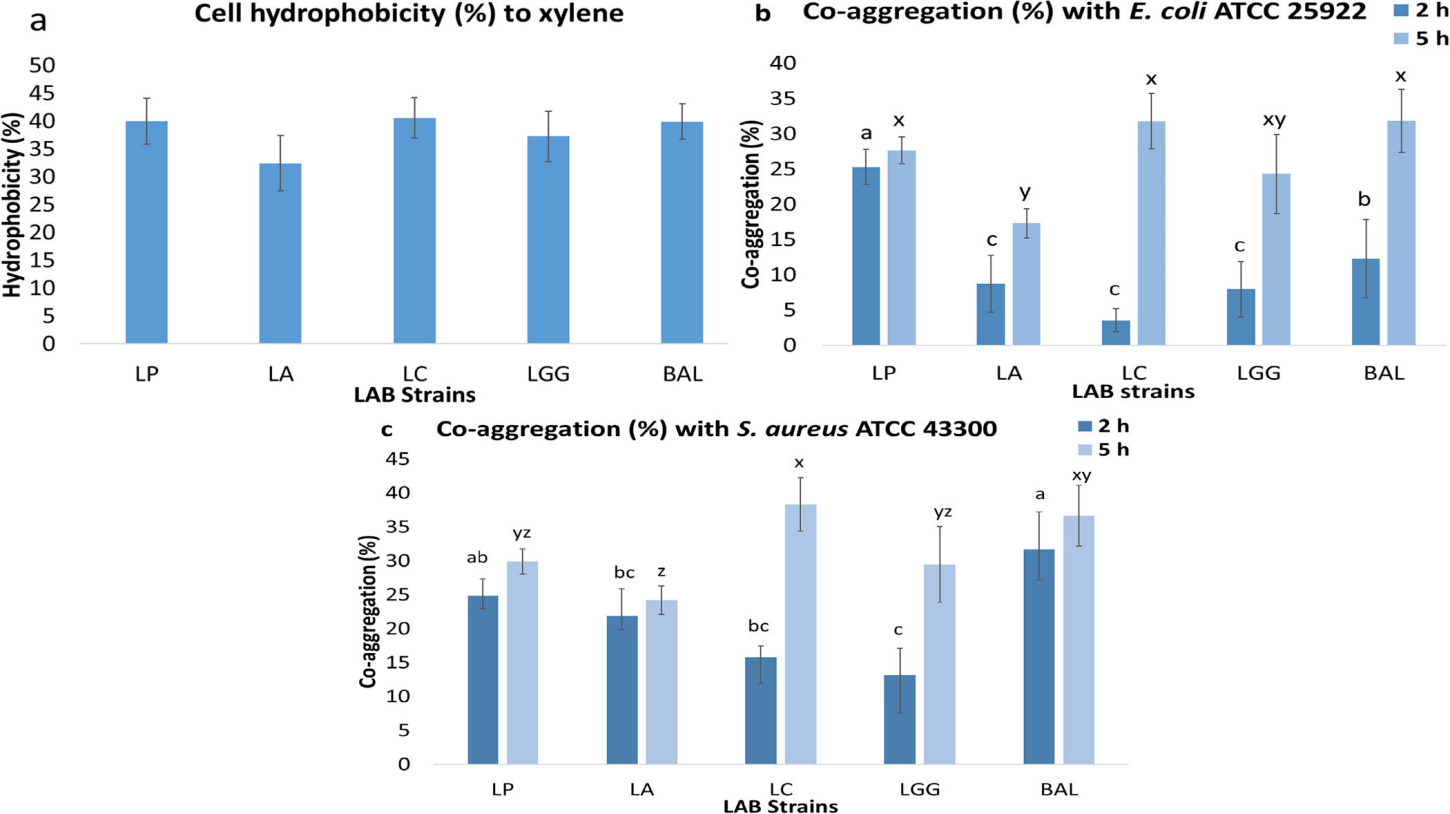
Cell surface properties of lactic acid bacteria (LAB) strains: (a) cell surface hydrophobicity (%) of LAB strains to xylene after 1 h of coincubation, (b) coaggregation (%) of LAB strains with *Escherichia coli* ATCC 25922 after 2 and 5 h of coincubation, and (c) coaggregation (%) of LAB strains with *Staphylococcus aureus* ATCC 43300 after 2 and 5 h of coincubation. The results shown are the means of three replications ± SD. Lowercase letters (a–c, x–z) represent statistically significant differences between the LAB strain (*n* = 3, *p* < 0.05, Duncan's test) after 2 and 5 h, respectively. LP, *Lactiplantibacillus plantarum* NBRC 3070; LA, *Lactobacillus acidophilus* ATCC 4356; LC, *Lacticaseibacillus casei* ATCC 393; LGG, *Lacticaseibacillus rhamnosus* GG ATCC 53103; BAL, *Bifidobacterium animalis* subsp. *lactis* ATCC 27673.

Nonsignificant differences (*p* > 0.05) in cell surface hydrophobicity were observed among the five LAB strains. Nonetheless, *L. plantarum* NBRC 3070, *L. casei* ATCC 393, and *B. animalis* subsp. *lactis* ATCC 27673 demonstrated numerically higher hydrophobicity values (~65%) compared with *L. acidophilus* ATCC 4356 and *L. rhamnosus* GG ATCC 53103, which showed lower values ranging from 59% to 62% (Figure 4a).

#### 3.1.5 Autoaggregation

The autoaggregation abilities of five LAB strains at different incubation times are presented in Table 3. All strains demonstrated time‐dependent increases in autoaggregation, with percentages ranging from 11.67% ± 1.42% to 31.22% ± 0.54% at 2 h, 17.49% ± 0.09% to 43.53% ± 0.13% at 5 h, and 76.44% ± 2.65% to 91.36% ± 0.74% at 24 h. After 5 h of incubation, *B. animalis* subsp. *lactis* ATCC 27673 (43.53% ± 0.13%), *L. rhamnosus* GG ATCC 53103 (37.77% ± 1.39%), and *L. plantarum* NBRC 3070 (35.97% ± 0.78%) showed the highest autoaggregation levels. At 24 h, *L. casei* ATCC 393, *L. rhamnosus* GG ATCC 53103, and *B. animalis* subsp. *lactis* ATCC 27673 achieved autoaggregation values above 85%, which were significantly higher (*p* < 0.05) than those of other strains. In contrast, *L. acidophilus* ATCC 4356 consistently displayed lower autoaggregation potential throughout the incubation period.

**Table 3:** Autoaggregation (%) of lactic acid bacteria (LAB) strains after incubating 2, 5, and 24 h at 37°C.

Lactic acid bacteria	Strains	Incubation periods		
		2 h	5 h	24 h
*Lactiplantibacillus plantarum*	NBRC 3070	31.22 ± 0.54^a^	35.97 ± 0.78^b^	81.45 ± 2.15^c^
*Lactobacillus acidophilus*	ATCC 4356	11.67 ± 1.42^b^	17.49 ± 0.09^d^	76.44 ± 2.65^c^
*Lacticaseibacillus casei*	ATCC 393	14.73 ± 1.66^b^	28.45 ± 2.69^c^	89.70 ± 1.06^ab^
*Lacticaseibacillus rhamnosus* GG	ATCC 53103	16.30 ± 0.78^b^	37.77 ± 1.39^b^	91.36 ± 0.74^a^
*Bifidobacterium animalis* subsp. *lactis*	ATCC 27673	29.17 ± 5.21^a^	43.53 ± 0.13^a^	84.89 ± 2.60^b^
SEM		1.481	0.810	1.158
*p* value		0.000	0.000	0.000

*Note:* The results shown are the means of three replications ± SD. Lowercase letters (a, b, c, and d) represent statistically significant differences among different LAB strains within each incubation time (h) (*n* = 3, *p* < 0.05, Duncan's test).

Abbreviation: SEM, standard error of mean.

#### 3.1.6 Coaggregation

The coaggregation potential of five LAB strains with *S. aureus* ATCC 43300 and *E. coli* ATCC 25922 are presented in Figure 4b,c and Table S5 for 2 and 5 h at 37°C. At 5 h, *L. casei* ATCC 393 and *B. animalis* subsp. *lactis* ATCC 27673 exhibited the highest coaggregation potential (*p* < 0.01) with *E. coli* ATCC 25922 (approximately 32%) and *S. aureus* ATCC 43300 (36%–38%). Other LAB strains showed lower coaggregation levels, ranging from 17.31% to 27.63% for *E. coli* ATCC 25922 and 24.19% to 29.88% for *S. aureus* ATCC 43300 at 5 h of incubation. Overall, LAB strains demonstrated higher coaggregation potential with Gram‐positive *S. aureus* (average 31.70%) than with Gram‐negative *E. coli* (average 26.97%). A correlation analysis (Table 4) revealed a nonsignificant (*p* > 0.05) positive relationship between surface hydrophobicity and autoaggregation, while significant positive correlations (*p* < 0.05) were observed between both surface hydrophobicity and autoaggregation with coaggregation toward *E. coli* ATCC 25922 and *S. aureus* ATCC 43300. These findings suggest that strains with stronger hydrophobic surfaces and self‐aggregation tendencies are also more capable of coaggregating with pathogenic bacteria, supporting their potential adhesion and competitive exclusion abilities, key attributes for probiotics potential. However, the coaggregation potential of *Lactobacillus* spp. varied significantly depending on the indicator strains (*p* < 0.01).

**Table 4:** Pearson correlation coefficient between cell surface hydrophobicity, autoaggregation, and coaggregation of all probiotic lactic acid bacteria strains.

Parameters	Autoaggregation	Coaggregation		Surface hydrophobicity
		*Escherichia coli* ATCC 25922	*Staphylococcus aureus* ATCC 43300	
Autoaggregation	1			
Coaggregation				
*E. coli ATCC 25922*	0.590* (*p* < 0.05)	1		
*S. aureus* ATCC 43300	0.479 (*p* > 0.05)	0.820** (*p* < 0.01)	1	
Surface hydrophobicity	0.512 (*p* > 0.05)	0.585* (*p* < 0.05)	0.614* (*p* < 0.05)	1

* Correlation is significant at 0.05 (two‐tailed, Pearson correlation).

** Correlation is significant at 0.01 (two‐tailed, Pearson correlation).

### 3.2 Cumulative Growth and Production of AMSs From LAB Strains

**Figure 5:**
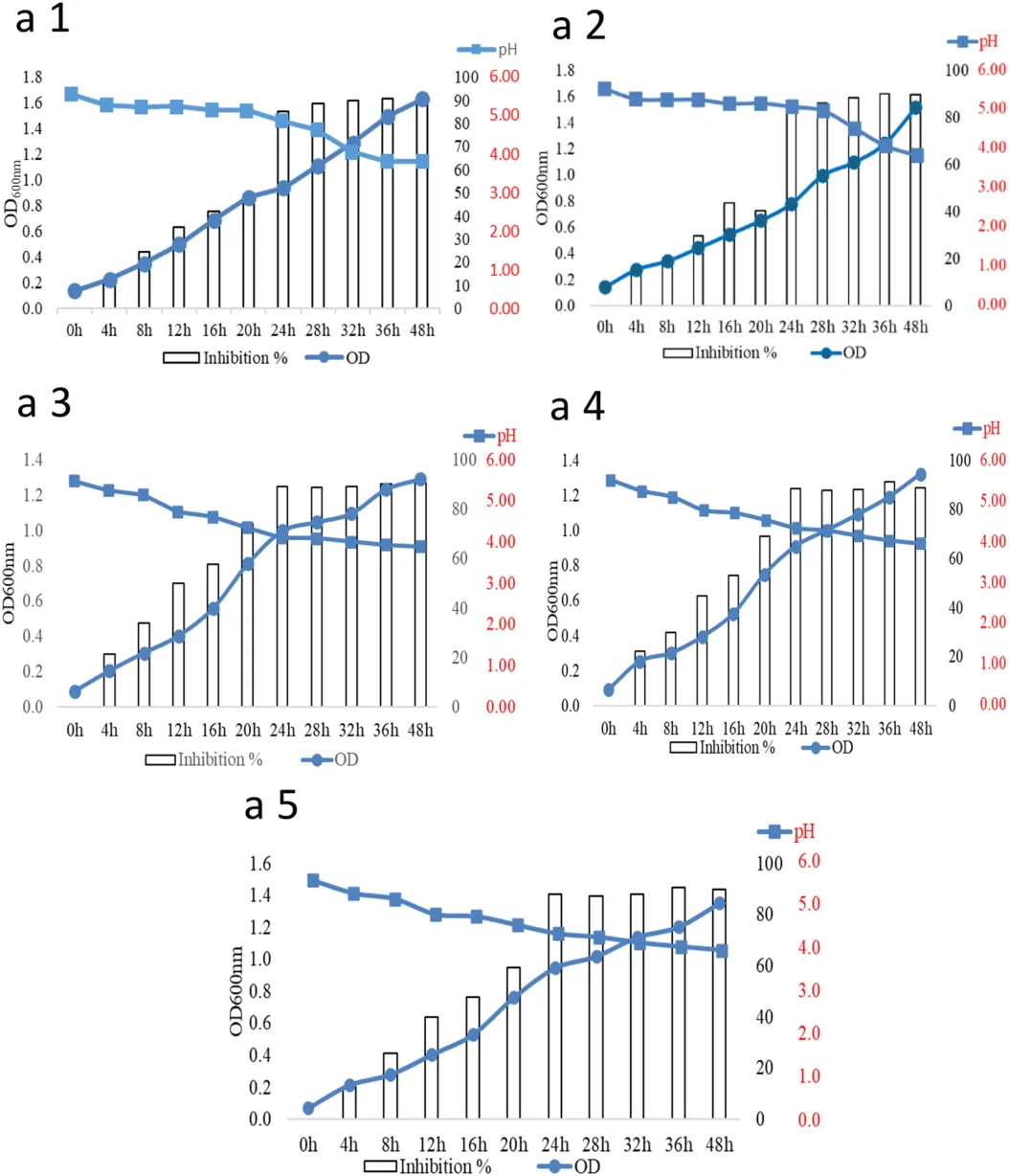
Cell density (OD values), pH, and antimicrobial activities (% inhibition) of inhibitory substances in CFS of (1) *Lactiplantibacillus plantarum* NBRC 3070, (2) *Lactobacillus acidophilus* ATCC 4356, (3) *Lacticaseibacillus casei* ATCC 393, (4) *Lacticaseibacillus rhamnosus* GG ATCC 53103, and (5) *Bifidobacterium animalis* ATCC 27673 strains under different growth conditions of 30°C (a). The growth of LAB strains was determined spectrophotometrically at OD_600nm_ (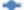). While line graphs (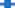) and bars (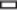) indicate the pH in MRS broth and the antimicrobial activity of BLIS in LAB strains. The antimicrobial spectrum was determined by broth microdilution assay at three sampling times, where *Staphylococcus aureus* ATCC 43300 was used as an indicator bacterium. BLIS, bacteriocin‐like inhibitory substance; CFS, cell‐free supernatant; LAB, lactic acid bacteria; MRS, de Man, Rogosa, and Sharpe; OD, optical density.

**Figure 6:**
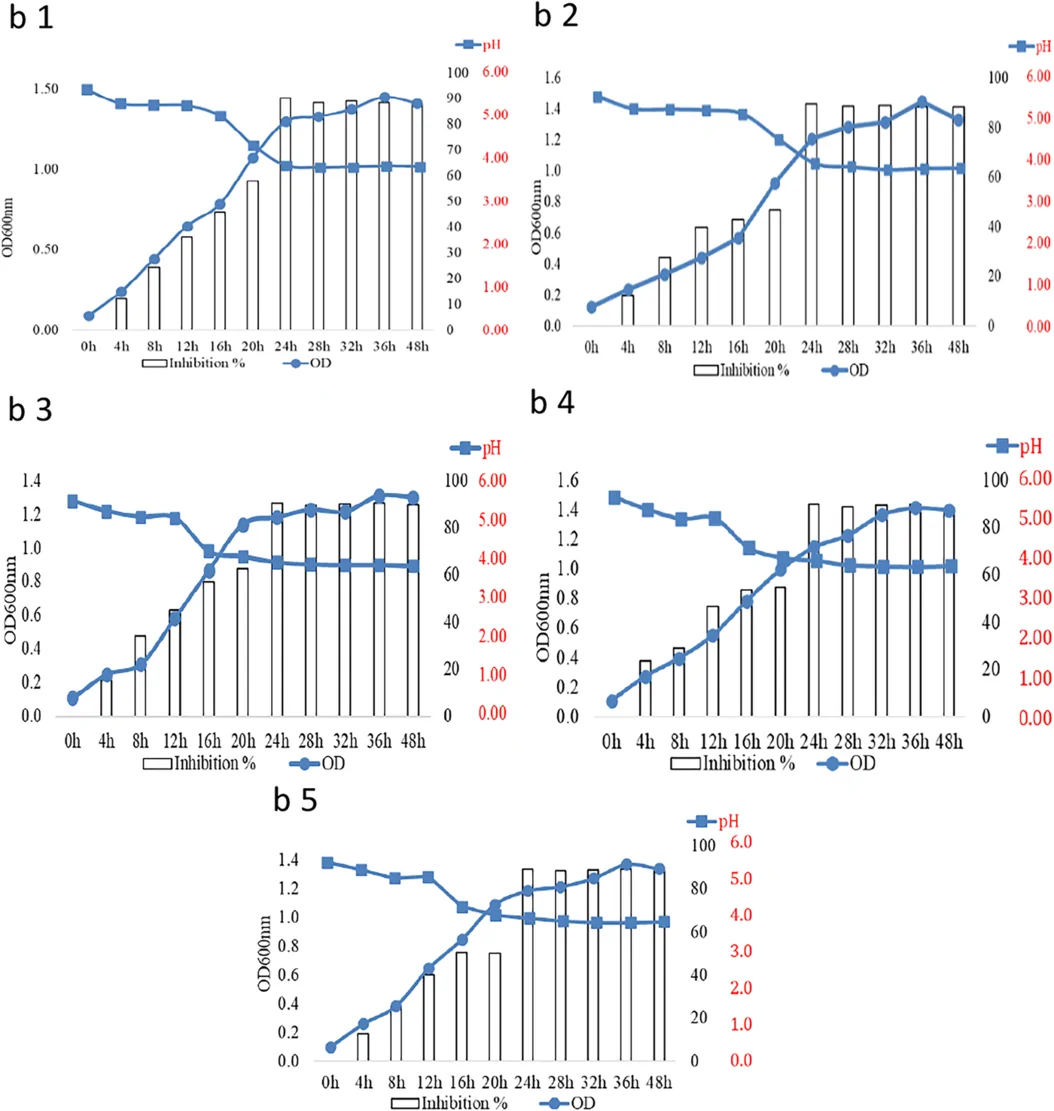
Cell density (OD values), pH, and antimicrobial activities (% inhibition) of inhibitory substances in CFS of (1) *Lactiplantibacillus plantarum* NBRC 3070, (2) *Lactobacillus acidophilus* ATCC 4356, (3) *Lacticaseibacillus casei* ATCC 393, (4) *Lacticaseibacillus rhamnosus* GG ATCC 53103, and (5) *Bifidobacterium animalis* ATCC 27673 strains under different growth conditions of 37°C (b). The growth of LAB strains was determined spectrophotometrically at OD_600nm_ (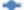). While line graphs (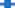) and bars (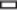) indicate the pH in MRS broth and the antimicrobial activity of BLIS in LAB strains. The antimicrobial spectrum was determined by broth microdilution assay at three sampling times, where *Staphylococcus aureus* ATCC 43300 was used as an indicator bacterium. BLIS, bacteriocin‐like inhibitory substance; CFS, cell‐free supernatant; LAB, lactic acid bacteria; MRS, de Man, Rogosa, and Sharpe; OD, optical density.

LAB strains were cultured in MRS broth (initial pH 6.20 ± 0.2) for 0–48 h at 30°C and 37°C. Growth and cumulative production of AMS were monitored (Figures 5 and 6). At both temperatures, the initial AMS activity (11.90%–23.61%) was observed as early as 4 h during the initial logarithmic growth phase. This was accompanied by the pH values of 5.51–5.57 and low cell biomass (OD_600nm_, 0.21–0.28). The maximum AMS production (over 90% inhibition) occurred during the late stationary phase (36 h) at 30°C (*p* < 0.01), while at 37°C, a similar peak (≥ 89% inhibition) was reached earlier at 24 h. At both temperatures, this peak AMS activity was associated with a drop in pH to 3.82–4.05 and higher cell biomass (OD_600nm_, 1.15–1.52). The AMS production remained stable between 24 and 36 h (85.36%–91.29%) before gradually decreasing. At 30°C, LAB strains showed a prolonged exponential phase from 28 to 48 h (Figure 5), while at 37°C, growth plateaued earlier (24 h), followed by a decline after 36 h (Figure 6). Notably, *L. acidophilus* ATCC 4356 consistently exhibited the slowest growth rate, as reflected in lower OD values. Interestingly, increased incubation temperature (37°C) accelerated bacterial growth but did not proportionally enhance AMS production; however, it was strongly correlated with a decline in pH and biomass accumulation. The main effect of incubation time had a statistically significant influence on AMS production (*p* < 0.01), as did LAB strains differences (*p* < 0.05). Additionally, two‐way and three‐way interactions between incubation time, temperature, and strains were also significant (two‐way, *p* < 0.01 and three‐way, *p* < 0.05), as summarized in Table 5. However, temperatures alone did not significantly affect AMS levels (*p* = 0.631). Furthermore, detailed analysis of the effects of incubation conditions on active AMS production, growth, and pH changes is presented in supporting materials (Tables S6, S7, and S8).

**Table 5:** The effect of different incubation temperatures (30°C and 37°C) and times (12, 24, 36, and 48 h) of incubating different LAB strains on their cell growth (OD_600nm_), pH values, and the inhibition percentages of CFS obtained from LAB strains.

ANOVA (*p* value)	Cell growth (OD_600nm_)	pH values	Inhibition percentage (%)
*Main effects*			
Incubation temperature (°C)	0.000	0.000	0.631
Incubation time (*H*)	0.000	0.000	0.000
Treatment (*T*)	0.029	0.003	0.017
*Two‐way interactions*			
°C × *H*	0.000	0.000	0.000
°C × *T*	0.024	0.000	0.000
*H* × *T*	0.000	0.000	0.000
*Three‐way interactions*			
°C × *H* × *T*	0.000	0.000	0.022

Abbreviations: ANOVA, analysis of variance; CFS, cell‐free supernatant; LAB, lactic acid bacteria; OD, optical density.

### 3.3 Quantification of AMSs Activity of LAB Strains

**Figure 7:**
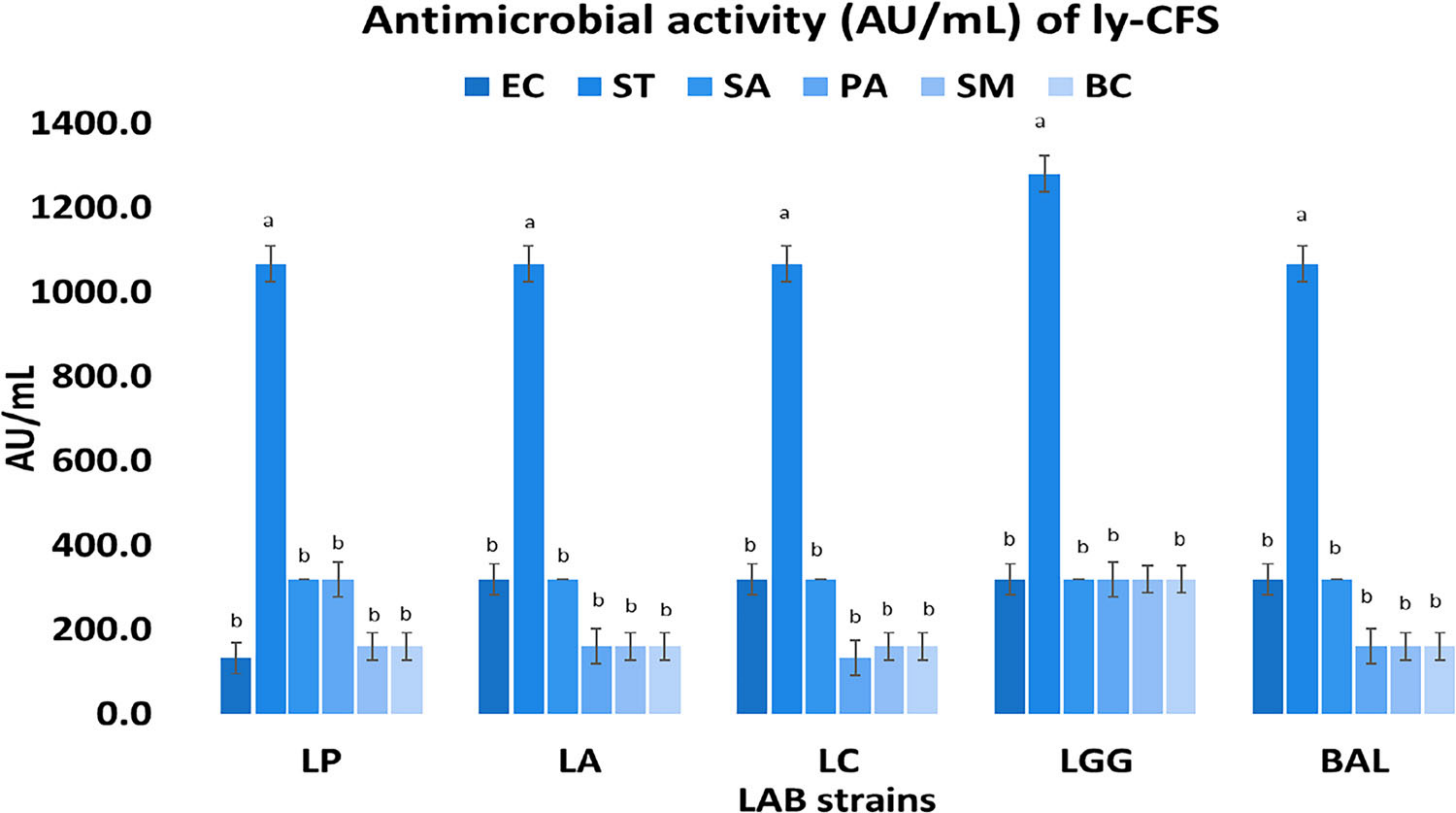
Antimicrobial activity (AU/mL) of ly‐CFS containing antibacterial substances obtained from five LAB strains against four pathogenic bacteria. Lowercase letters (a and b) represent statistically significant differences in LAB strains tested between the pathogenic strain (*n* = 3, *p* < 0.05, Duncan's test). EC, *Escherichia coli* ATCC 25922; ST, *Salmonella enterica serovar* Typhimurium ATCC 14028; SA, *Staphylococcus aureus* ATCC 43300; PA, *Pseudomonas aeruginosa* ATCC 10145; SM, *Serratia marcescens* ATCC 14756; BC, *Bacillus cereus* ATCC 14579; LP, *Lactiplantibacillus plantarum* NBRC 3070; LA, *Lactobacillus acidophilus* ATCC 4356; LC, *Lacticaseibacillus casei* ATCC 393; LGG, *Lacticaseibacillus rhamnosus* GG ATCC 53103. The error bars indicate the standard error of the mean (*n* = 3). AU, arbitrary unit; LAB, lactic acid bacteria; ly‐CFS, lyophilized cell‐free supernatant.

The antimicrobial activities of the concentrated CFS (ly‐CFS) containing AMS from five LAB strains were quantified using AU/mL and MIC (mg/mL) values (Figure 7 and Table 6). The activity ranged from 133.33 to 1280 AU/mL across six indicator pathogens, with significant differences (*p* < 0.05) among the LAB strains and target organisms. *L. rhamnosus* GG ATCC 53103 exhibited the strongest overall antimicrobial activity, particularly against *S. Typhimurium* ATCC 14028. It achieved a maximum inhibition of 1280.00 AU/mL (*p* < 0.01) and an MIC of 1.54 mg/mL. Against other indicators, the inhibition was recorded at 320.00 AU/mL. Among the indicators, *S. Typhimurium* ATCC 14028 was strongly inhibited by the other four LAB strains, showing a value of 1066.00 AU/mL and an MIC of 3.13 mg/mL. Furthermore, the antibacterial activity of five LAB strains showed inhibition of 320.00 AU/mL against *S. aureus* ATCC 43300. In contrast, *L. acidophilus* ATCC 4356, *L. casei* ATCC 393, *B. animalis* subsp*. lactis* ATCC 27673 showed moderate activity (320.00 AU/mL) against *E. coli* ATCC 25922, while lower inhibitory values (133.67–166.67 AU/mL) were observed against *P. aeruginosa* ATCC 10145, *S. marcescens* ATCC 14756, and *B. cereus* ATCC 14579. The AMS from five LAB strains showed higher MIC values (1.54–12.5 mg/mL), suggesting that graded resistance in the pathogens tested. Conversely, *E. coli* ATCC 25922, *S. aureus* ATCC 43300, and *P. aeruginosa* ATCC 10145 were less affected, with MICs ranging from 6.26 to 12.5 mg/mL, especially for non‐*L. rhamnosus* strains.

**Table 6:** The inhibitory activity (%) in different concentrations of antibacterial agents is obtained from five LAB strains and the minimum inhibitory concentrations (MIC_90_) against six indicator strains.

LAB strains	Concentration (mg/mL)	MIC_90_ against pathogenic bacterial strains											
		*Escherichia coli*	MIC value (mg/mL)	*Salmonella enterica serovar* Typhimurium	MIC value (mg/mL)	*Staphylococcus aureus*	MIC value (mg/mL)	*Pseudomonas aeruginosa*	MIC value (mg/mL)	*Serratia marcescens*	MIC value (mg/mL)	*Bacillus cereus*	MIC value (mg/mL)
LP	50	++		++		++		++		++		++	
	25	++		++		++		++		++		++	
	12.5	++	12.5	++		++	12.5	++	12.5	++	12.5	++	12.5
	6.26	—		++		+		+		+		+	
	3.13	—		++	3.13	—		—		—		—	
	1.54	—		+		—		—		—		—	
LA	50	++		++		++		++		++		++	
	25	++		++		++		++		++		++	
	12.5	++		++		++	12.5	++	12.5	++	12.5	++	12.5
	6.26	++	6.26	++		+		—		+		+	
	3.13	+		++	3.13	—		—		—		—	
	1.54	—		+		—		—		—		—	
LC	50	++		++		++		++		++		++	
	25	++		++		++		++		++		++	
	12.5	++		++		++	12.5	++	12.5	++	12.5	++	12.5
	6.26	++	6.26	++		+		—		+		+	
	3.13	+		++	3.13	—		—		—		—	
	1.54	—		+		—		—		—		—	
LGG	50	++		++		++		++		++		++	
	25	++		++		++		++		++		++	
	12.5	++		++		++		++	12.5	++		++	
	6.26	++	6.26	++		++	6.26	+		++	6.26	++	6.26
	3.13	—		++		—		—		—		—	
	1.54	—		++	1.54	—		—		—		—	
BAL	50	++		++		++		++		++		++	
	25	++		++		++		++		++		++	
	12.5	++	12.5	++		++	12.5	++	12.5	++	12.5	++	12.5
	6.26	+		++		+		—		+		+	
	3.13	—		++	3.13	—		—		—		—	
	1.54	—		+		—		—		—		—	

*Note:* ++, ≥ 90% inhibition; +, ≥ 50% inhibition and < 50% inhibition; LP, *Lactiplantibacillus plantarum* NBRC 3070; LA, *Lactobacillus acidophilus* ATCC 4356; LC, *Lacticaseibacillus casei* ATCC 393; LGG, *Lacticaseibacillus rhamnosus* ATCC 53103, and BAL, *Bifidobacterium animalis* subsp. *lactis* ATCC 27673.

Abbreviation: LAB, lactic acid bacteria.

### 3.4 Characterization of AMSs Obtained From LAB Strains

#### 3.4.1 Characterization of CFS for BLIS Production

**Figure 8:**
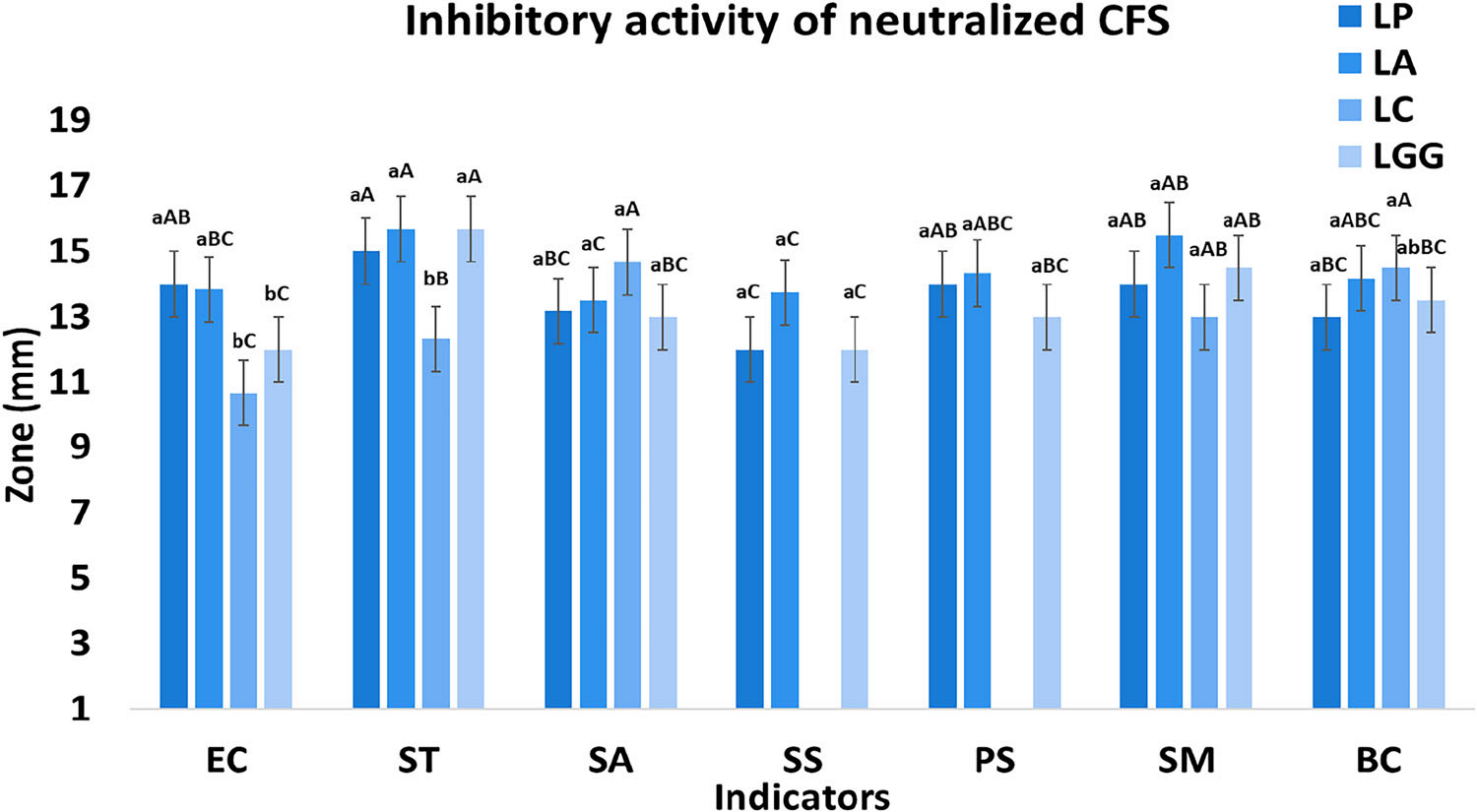
Diameter of inhibition zone (mm) obtained from n‐CFS of four LAB strains against seven indicators incubated for 24 h anaerobically by well diffusion assay. The error bars indicate the standard error of the mean (*n* − 3). EC, *Escherichia coli* ATCC 25922; ST, *Salmonella enterica serovar* Typhimurium ATCC 14028; SA, *Staphylococcus aureus* ATCC 43300; SS, *Shigella sonnei* ATCC 25931; PA, *Pseudomonas aeruginosa* ATCC 10145; SM, *Serratia marcescens* ATCC 14756; BC, *Bacillus cereus* ATCC 14579; LP, *Lactiplantibacillus plantarum* NBRC 3070; LA, *Lactobacillus acidophilus* ATCC 4356; LC, *Lacticaseibacillus casei* ATCC 393; LGG, *Lacticaseibacillus rhamnosus* GG ATCC 53103. Lowercase letters (a, b, c, and d) represent statistically significant differences among different LAB strains within each indicator strain. Uppercase letters (A, B, and C) represent statistically significant differences among different indicator strains within each LAB strain (*n* = 3, *p* < 0.05, Duncan's test). LAB, lactic acid bacteria; n‐CFS, neutralized cell‐free supernatant.

The inhibition zones of ly‐CFS from all LAB strains against four indicator bacteria are shown in Figure 8. Apart from *B. animalis* subsp. *lactis* ATCC 27673, the ly‐CFS of the other four strains, maintained antimicrobial activity, demonstrating the presence of BLIS. The lack of BLIS activity in *B. animalis* subsp. *lactis* ATCC 27673 suggests that its inhibition may be primarily due to organic acid or other pH‐sensitive compounds rather than stable BLIS. The moderate antimicrobial activity of four LAB strains ranged from 10 ± 0.57 to 15.83 ± 0.28 mm, only confirming that the activity is due to the BLIS. Significant differences (*p* < 0.05) in BLIS activity were observed among the four LAB strains against *E. coli* ATCC 25922, *S. Typhimurium* ATCC 14028, and *B. cereus* ATCC 14579. Conversely, *P. aeruginosa* ATCC 10145 and *S. sonnei* ATCC 25931 showed resistance to BLIS of *L. casei* ATCC 393. Overall, BLIS activity in ly‐CFS was found significantly lower (*p* < 0.01) compared with r‐CFS (AMS) activity, with reductions ranging from 11.34% to 100% (Figure S1 and Table S9).

#### 3.4.2 Enzymatic Characterization of Bacteriocins‐Like Inhibitory Substance

**Figure 9:**
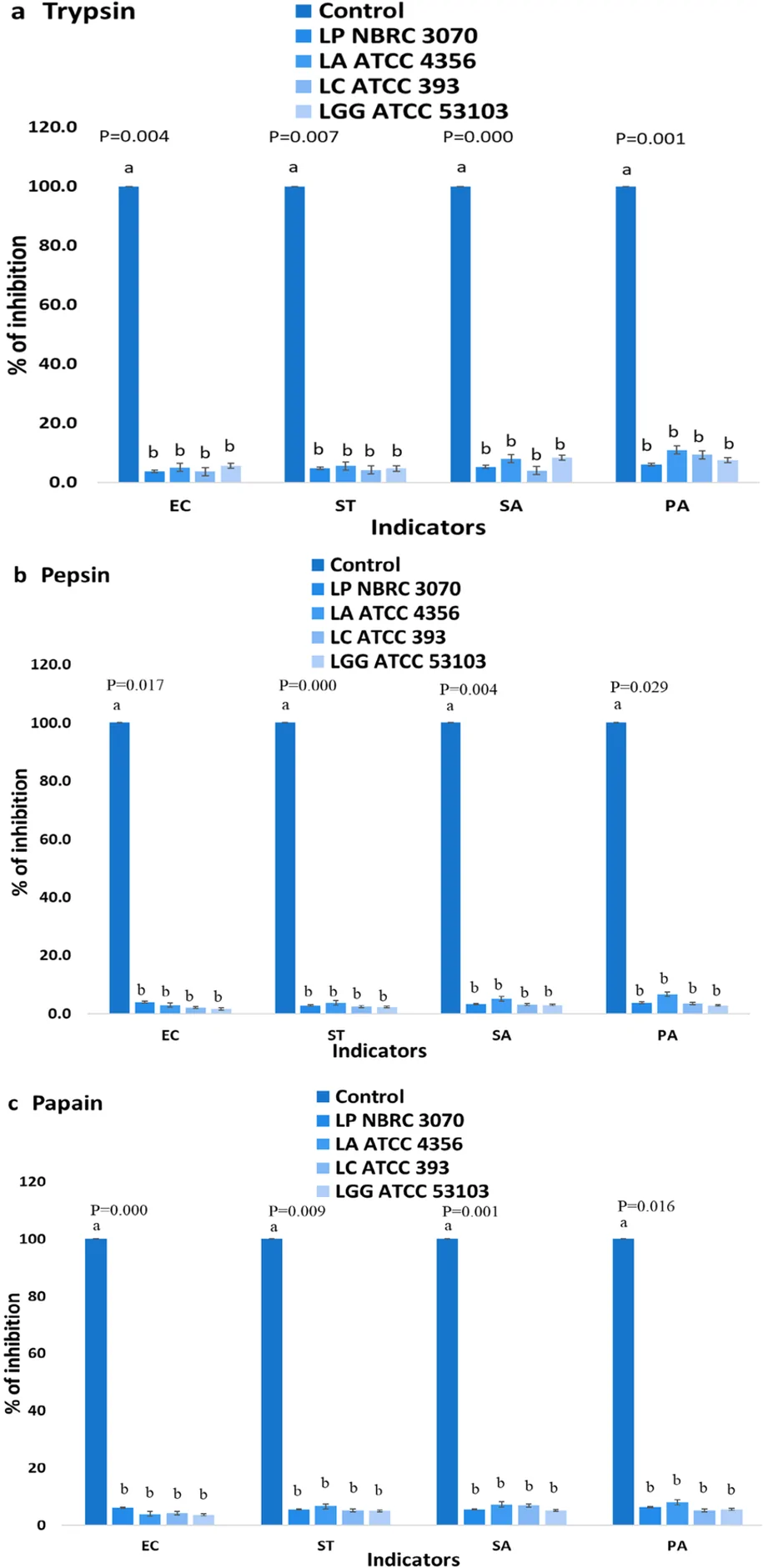
Inhibition activity (%) obtained from ly‐CFS of four LAB strains treated with (a) trypsin, (b) pepsin, (c) papain in broth microdilution assay against *Escherichia coli* ATCC 25922 (EC), *Salmonella enterica serovar* Typhimurium ATCC 14028 (ST), *Staphylococcus aureus* ATCC 43300 (SA), and *Pseudomonas aeruginosa* ATCC 10145 (PA) for 24 h anaerobically. The inhibition activity of enzyme‐treated ly‐CFS against indicator strains was compared with positive control (100% activity of CFS) cultures, with three replications. The error bars indicate the standard error of the mean (*n* = 3). Lowercase letters (a and b) represent statistically significant differences within each indicator strain among the BLIS of the LAB strain (*n* = 3, *p* < 0.05, Duncan's test). CFS, cell‐free supernatant; LAB, lactic acid bacteria; ly‐CFS, lyophilized cell‐free supernatant.

The antibacterial activity of the concentrated CFS (ly‐CFS) from four LAB strains significantly decreased (*p* < 0.05) after treatment with proteolytic enzymes, trypsin (Figure 9a), pepsin (Figure 9b), and papain (Figure 9c), compared with the untreated control (defined as 100% activity). The observed reduction ranged from 1.28% to 10.97%, indicating that the active compounds are likely proteinaceous in nature, consistent with characteristics of BLIS. Among the enzymes, numerically, the pepsin‐treated ly‐CFS showed a pronounced decrease (average 3.34%) in BLIS activity compared with trypsin (6.07%) and papain (5.59%). However, the ly‐CFS from the LAB strains did not show a significant difference (*p* > 0.05) in antimicrobial activity against the indicator strains.

## 4 Discussion

This study evaluated the probiotic potential and antimicrobial efficacy of five LAB strains based on their inhibition of foodborne and spoilage pathogens. These strains were chosen for their established probiotic properties and antimicrobial metabolites production, such as organic acid, bacteriocins, and BLIS (Abouloifa et al. 2022; Echegaray et al. 2023). Initial screening using the WBC demonstrated variable inhibitory activity against a broad range of pathogens of *E. coli*, *S. Typhimurium*, *S. aureus*, *S. sonnei*, *P. aeruginosa*, *S. marcescens*, and *B. cereus*. These findings confirm that LAB exerts antimicrobial effects via secreted metabolites and potential cell contact‐dependent mechanisms of Gram‐positive and Gram‐negative pathogenic bacteria (Wasfi et al. 2018; Ołdak et al. 2020).

Following this, the r‐CFS containing postbiotics were assessed and exhibited significant inhibition (*p* < 0.01), especially Gram‐negative strains, which aligns with previous findings (Bungenstock et al. 2020; R. Du et al. 2022). Notably, *L. plantarum* and *L. casei* revealed the most potent activity, with inhibition zones > 20 mm, classifying the pathogens as extremely sensitive (Moradi et al. 2021; Yang et al. 2024). This strong antimicrobial effect likely results from the combined action of AMS, including bacteriocins, organic acids, and diacetyl, which disrupts the outer membrane of Gram‐negative bacteria (Yi et al. 2020; Tavares‐Carreon et al. 2023). These results indicate the potential of *L. plantarum* and *L. casei* as natural food biopreservatives by improving safety and extending the shelf life. Our results corroborated earlier findings of Arrioja‐Bretón et al. (2020), Thuy et al. (2024), and Divyashree et al. (2021) where CFS of *L. plantarum* (strains NRRL B‐4496 and CYLB47) and *L. casei* MYSRD exhibited the highest inhibition against Gram‐negative (*Salmonella* spp*., Pseudomonas* spp., and *E. coli)* than Gram‐positive (*S. aureus*) bacteria. In the present study, *L. rhamnosus* GG and *L. acidophilus* effectively inhibit *S. aureus*, making them key strains for preventing food contamination associated with this bacterium. Additionally, *S. sonnei* showed the least sensitivity to all LAB‐derived r‐CFS, with zones measuring 15–18 mm. This may stem from the strain‐specific resistance traits or potentially due to resistance mechanisms or reduced membrane permeability to AMS (Shad and Shad 2021).

Broth microdilution confirmed these findings, showing a high inhibition percentage (84%–89%) against *E. coli, S. Typhimurium*, and *S. aureus*, especially from *L. plantarum* and *L. casei* postbiotics. This reinforced their effectiveness in pathogen control in both food production and processing. The variability in inhibition observed across methods and stains is likely due to differences in AMS concentrations, pathogen type, and detection method used (Balouiri et al. 2016; Cizeikiene and Jagelaviciute 2021). *L. plantarum* was the most broadly effective strain, followed by *L. rhamnosus* GG and *L. casei*. WBC generally displayed more pronounced activity than r‐CFS, due to the synergistic effect of live cells and their metabolite production (Ołdak et al. 2020). This observation highlights the importance of considering the application approaches of LAB in food systems, either live cells directly or their AMS, to achieve optimal antimicrobial activity.

Antibiotic susceptibility is a critical safety criterion for probiotics, especially when intended for food applications. The EFSA emphasizes the need to screen probiotic strains for antibiotic resistance (AMR) to avoid the risk of transferring AMR genes to gut bacteria (Dobreva et al. 2022). However, LAB may have resistance to ciprofloxacin, gentamicin, streptomycin, and tetracycline, but this resistance is generally not transferable and does not pose a risk when using probiotics (Jose et al. 2015). Antibiotic susceptibility testing showed that most LAB strains were sensitive to commonly used antibiotics, supporting their safe use in food systems. In this study, most strains were sensitive to azithromycin, tetracycline, ciprofloxacin, and penicillin, which is consistent with previous findings (Rossi et al. 2015; Nunziata et al. 2022). However, all strains exhibited resistance to aminoglycosides like streptomycin and gentamicin, and to norfloxacin, an outcome also observed in earlier studies and attributed to intrinsic, nontransferable resistance mechanisms typical of LAB (Anisimova and Yarullina 2019). However, in contrast to the current findings, Nunziata et al. (2022) and Ali et al. (2020) reported the sensitivity of LAB strains to streptomycin and gentamicin. Complete resistance in *L. plantarum* to all antibiotics tested warrants further investigation for AMR gene presence. This aligns with the results reported by Dobreva et al. (2022) and may be due to either inherent resistance traits or environmental adaptation. In contrast, *L. rhamnosus* GG displayed sensitivity to multiple antibiotic classes, including β‐lactams, tetracyclines, and macrolides. This is consistent with the findings of Ali et al. (2020), confirming its safety profile and supporting its application in food and therapeutic contexts. The resistance variations of *L. acidophilus*, *L. casei*, and *B. animalis* subsp. *lactis* to azithromycin, ciprofloxacin, and penicillin underscore the strain‐specific natures of LAB (Ojha et al. 2023; Ali et al. 2020).

*Lactobacillus* spp. possesses surface characteristics that allow it to colonize the gastrointestinal tract and act as biopreservatives, helping it adhere to cells, compete with pathogens, and survive harsh conditions (Meng et al. 2018). This study evaluated cell surface properties of LAB strains, such as hydrophobicity, autoaggregation, and coaggregation for probiotic potential. *L. plantarum, L. casei*, and *B. animalis* subsp. *lactis* exhibited greater hydrophobicity (> 65%), correlating with strong intestinal adhesion potential (Yang et al. 2024). Previous studies have demonstrated wide variability in hydrophobicity with xylene (6%–96.62%) among LAB strains, depending on the strains, assay method, and environmental conditions (Chantanawilas et al. 2024; Singh et al. 2021; Cai et al. 2022). In contrast, other findings reported over 95% hydrophobicity of *Lactobacillus* spp. with xylene (A. Sharma et al. 2021; Sahoo et al. 2015). All strains in this study were classified as “strongly hydrophobic” (> 50%), indicating favorable adhesion characteristics due to the presence of hydrophobic molecules like proteins and lipids (Tyfa et al. 2015; H. Panda et al. 2017). Autoaggregation is another key probiotic trait that facilitates colonization, which indicates a connection between genetically identical microorganisms and their adherence to host cells (Beldarrain‐Iznaga et al. 2021). In this study, autoaggregation of LAB strains increased over time, with *L. rhamnosus* GG and *L. casei* reaching the highest levels (> 85%) at 24 h, indicating strong colonizing ability through hydrophobic interactions of exopolysaccharides, lipoteichoic acid, and surface‐layer proteins (Nwoko and Okeke 2021). These levels surpass or align with earlier findings of Piwat et al. (2015) and Sophatha et al. (2020), confirming that high autoaggregation enhances probiotic persistence and inhibits the pathogen colonization property of LAB strains (Bhat and Bajaj 2020; J. Lee, Jo, et al. 2024). The highest autoaggregation of the *L. plantarum* reached 31.22% after 2 h, within the previously reported range of 8.4%–59% (Mohanty et al. 2019; Zawistowska‐Rojek et al. 2022), and *L. rhamnosus* GG reached 43.53% at 5 h, which is comparatively lower than the 47%–92% observed in earlier studies (Bhat and Bajaj 2020; Yang et al. 2024).

The coaggregation abilities of probiotic strains can impede the ability of pathogenic strains to infect the host and hinder the colonization of foodborne pathogens (Chantanawilas et al. 2024). Coaggregation capacity was moderate but strain‐dependent, with *L. casei* and *B. animalis* subsp. *lactis* showing the highest interaction with both Gram‐positive (*S. aureus*) and Gram‐negative (*E. coli*) pathogens. Previous studies reported that *Lactobacillus* spp. exhibited 18%–77% coaggregation with pathogens such as *E. coli, S. aureus, Campylobacter* spp*., B. cereus*, and *Micrococcus* spp. exceeding the current findings (Cai et al. 2022; Bhat and Bajaj 2020). A strong positive correlation between autoaggregation and coaggregation confirms that strains with strong self‐adherence may be better equipped to coaggregate with the gut microbiota and inhibit pathogens (Chantanawilas et al. 2024; J. Lee, Jo, et al. 2024). Altogether, the strong antimicrobial, safety, and colonizing traits of LAB strains affirm their potential application in functional food systems and as natural alternatives to chemical preservatives. Temperature significantly impacts AMS production by influencing biosynthesis genes and the structural integrity (Wei and Zhang 2022). This study evaluated the impact of incubation temperatures (30°C vs. 37°C) and time (0–48 h) on AMS production and biomass accumulations by five LAB strains in MRS broth. AMS production was closely linked to the bacterial growth phase, pH decline, and cell density (OD_600nm_), with detectable inhibitory activity (11.9%–23.6%) as early as 4 h during the logarithmic phase, suggesting AMS may act as both a primary and secondary metabolite (Dai et al. 2022). Peak AMS production was observed during the early stationary phase at 37°C and the late stationary phase at 30°C, exceeding > 90% inhibition, before declining, indicating a time‐limited production window. Faster growth at 37°C resulted in quicker acidification and earlier OD_600nm_ peaks compared with 30°C, supporting the notion that low pH and nutrient stress trigger AMS biosynthesis (Mani‐López et al. 2022; Sionek et al. 2024). *L. acidophilus* showed slower growth at both temperatures, possibly due to its homofermentative metabolism and preference for temperatures above 37°C (Terpou et al. 2019; Sionek et al. 2024). The decline in AMS activity after its peak may be due to proteolytic enzyme degradation or protein aggregation (Woraprayote et al. 2015). These results reinforce the growth‐phase dependence of AMS biosynthesis (Abbasiliasi et al. 2017; Kalhoro et al. 2019).

AMS activity was quantified using AUs and MIC via broth microdilution assay against four pathogens with a twofold serial dilution. The strongest AMS inhibitory activity of LAB strains was observed against *S. Typhimurium* (1066.67–1280.00 AU/mL and MIC of 1.54–3.13 mg/mL), highlighting the promising food safety role of AMS with their potent inhibitory effects, particularly in controlling *Salmonella* contamination. The present findings are notably lower than those reported by Evangelista et al. (2021), who noted MICs of 11.25 mg/mL for *L. rhamnosus* ATCC 7469, *L. plantarum* PUCPR 44, and *L. acidophilus* Llorente against *S. Typhimurium*. Additionally, MIC values against *E. coli*, *S. aureus*, and *P. aeruginosa* ranged from 6.26 to 12.5 mg/mL, indicating moderate activity, consistent with previous findings (J. Wang et al. 2023; Evangelista et al. 2021). In contrast, Koohestani et al. (2018) revealed that *L. acidophilus* LA5 and *L. casei* 431 supernatants had substantially higher MIC values (40 mg/mL) against *S. aureus* ATCC 25923, further emphasizing the comparatively stronger activity observed in this study. Variation in MIC likely reflects differences in AMS structures, concentration, and target pathogen defense mechanisms, such as protease production, biofilm formation, and membrane composition (Breijyeh et al. 2020; Tavares et al. 2020). AMS quantification revealed strain‐specific activity, with *L. rhamnosus* GG demonstrating significant inhibitory potential, supporting its potential as a natural preservative in food systems.

The ly‐CFS from four LAB strains retained strong antimicrobial activity, with inhibition zones ranging from 10.00 ± 0.57 to 15.83 ± 0.28 mm, confirming the presence of BLIS. Among the strains, *L. acidophilus* showed the strongest inhibition, suggesting its BLIS may have favorable structural properties or higher potency (Md Sidek et al. 2018; Thuy et al. 2024). Consistent with the studies of Da Silva et al. (2022) and Evangelista et al. (2021), which demonstrated the effectiveness of *L. acidophilus* BLIS against both Gram‐positive (*S. aureus*) and Gram‐negative (*S. Typhimurium*) pathogens. Additionally, BLIS of *L. plantarum* showed notable activity against *E. coli*, consistent with previous reports (Evangelista et al. 2021). In contrast, BLIS of *L. casei* exhibited weak inhibition against *P. aeruginosa*, possibly due to structural or compositional limitations in its BLIS, insufficient to overcome the defense mechanisms of pathogens (Qin et al. 2022). Proteolytic enzyme treatment (trypsin, pepsin, and papain) of BLIS reduced antimicrobial activity by 1.28%–10.97% (*p* < 0.05), verifying that BLIS activity is protein‐based. Pepsin caused the most activity loss, suggesting specific peptide sensitivity to enzyme cleavage (Ashaolu et al. 2023).

## 5 Conclusion

The rise of MDR foodborne and spoilage pathogens underscores the need for an alternative to natural antimicrobials. This study demonstrated the promising antimicrobial potential of five LAB strains, *L. plantarum*, *L. acidophilus*, *L. casei*, *L. rhamnosus* GG, and *B. animalis* subsp. *lactis*, against a spectrum of MDR pathogens. The LAB‐derived AMSs, including BLIS, exhibited a strong inhibitory effect, with most inhibition zones exceeding 15 mm. These LAB strains also demonstrated desirable probiotic properties, including cell surface properties, aggregation abilities, and acceptable antibiotic susceptibility. Optimal AMS production was achieved during the stationary growth phase under acidic conditions, highlighting the importance of temperature and time optimization for maximizing antimicrobial yield. Despite these encouraging findings, the study has several limitations. Antimicrobial activity was assessed only in vitro with a limited range of pathogens. The molecular identity and structure of BLIS were not fully characterized, and the mechanism of antibiotic resistance, especially in *L. plantarum*, remained unclear. Further research should focus on expanding pathogen coverage to include more industrially relevant strains, conducting in vivo validation in food models, performing molecular and genetic characterization of BLIS, and investigating the mechanisms of antibiotic resistance in *L. plantarum*. Overall, these LAB strains hold promise as natural biopreservatives and probiotics candidates for enhancing food safety and combating AMR.

## Author Contributions

**Md. Moklesur Rahman:** data curation, writing – original draft, conceptualization, formal analysis. **Awis Qurni Sazili:** conceptualization, data curation, supervision, visualization, writing – review and editing. **Siti Aqlima Ahmad:** conceptualization, supervision, visualization, writing – review and editing. **Khalilah Abdul Khalil:** conceptualization, supervision, visualization, writing – review and editing. **Mohammad Rashedi Ismail‐Fitry:** supervision, visualization. **Md. Sazedul Karim Sarker:** funding acquisition, project administration, visualization.

## Ethics Statement

None of the authors has conducted any research on humans or animals for this article.

## Conflicts of Interest

None declared.

## Supporting information


**Supporting Figure S1:** The radar diagram shows the reduction percentage (%) of the inhibitory effect (ZOI, mm) by neutralized CFS (n‐CFS) produced by four LAB strains compared to raw CFS (r‐CFS) against indicator strains. LP, *L. plantarum* NBRC 3070; LA, *L. acidophilus* ATCC 4356; LC, *L. casei* ATCC 393; LGG, *L. rhamnosus* GG ATCC 53103.

**Supporting Table S1:** The growth and nature of LAB and pathogenic bacteria strains used for antibacterial activity test.

**Supporting Table S2:** The pH values of r‐CFS and n‐CFS from LAB strains were obtained 24 hours ago and used to screen antimicrobial activity.

**Supporting Table S3:** Antibiotics used in the study, along with their class and mode of antibacterial action.

**Supporting Table S4:** Diameter (mm) of inhibition zones with well diffusion assay generated by whole bacterial cell cultures of studied LAB strains against selected indicator strains for 24 h of incubation.

**Supporting Table S5:** Co‐aggregation (%) of lactic acid bacteria (LAB) strains with *E. coli* ATCC 25922 and *S. aureus* ATCC 43300 after incubating for 2 and 5 h at 37°C.

**Supporting Table S6:** The variation in cell growth (OD_600nm_ value) of five LAB strains incubated for 48 h at 30 and 37^o^C.

**Supporting Table S7:** The change in pH value of five LAB strains incubated for 48 h at 30 and 37^o^C.

**Supporting Table S8:** The inhibitory potential (%inhibition) against *S. aureus* of the bioactive substances produced from five LAB strains incubated for 48 h at 30 and 37^o^C.

**Supporting Table S9:** The comparative antimicrobial activity of r‐CFS and BLIS (n‐CFS) of four LAB strains against four pathogenic bacteria.

## Data Availability

Experimental data and supporting materials in this article will be available upon request.
