# Supplementary material for: Evaluation of Probiotic Properties and Antimicrobial Substances Produced by Five Lactic Acid Bacteria Against Foodborne and Spoilage Pathogens
Source: Microbiologyopen. 2026 Sep 25;15(5):e70052. doi: 10.1002/mbo3.70052 (PMC13613172; doi:10.1002/mbo3.70052)
Supplement: Supplementary file 1 [file MBO3-15-e70052-s001.docx]

**Evaluation of probiotic properties and antimicrobial substances produced by five lactic acid bacteria against foodborne and spoilage pathogens**

**MicrobiologyOpen**

Md. Moklesur Rahman^1,6^, Awis Qurni Sazili^1,2^, Siti Aqlima Ahmad^3^, Khalilah Abdul Khalil^4^, Mohammad Rashedi Ismail-Fitry^1,5^, and Md. Sazedul Karim Sarker^6^

^1^Halal Products Development, Halal Product Research Institute, Universiti Putra Malaysia, 43400, UPM Serdang, Selangor Darul Ehsan, Malaysia; ^2^Faculty of Agriculture, Universiti Putra Malaysia, 43400, UPM Serdang, Selangor Darul Ehsan, Malaysia; ^3^Faculty of Biotechnology and Bimolecular Science, Universiti Putra Malaysia, 43400, UPM Serdang, Selangor Darul Ehsan, Malaysia; ^4^Faculty of Applied Science, Universiti Teknologi MARA, 40450 Shah Alam, Selangor, Darul Ehsan, Malaysia; ^5^Faculty of Food Science and Technology, Universiti Putra Malaysia, 43400, UPM Serdang, Selangor Darul Ehsan, Malaysia; ^6^Bangladesh Livestock Research Institute (BLRI), Savar, Dhaka, Bangladesh.

**Corresponding author:** Awis Qurni Sazili (awis@upm.edu.my). Mailing address: Director, Halal Product Research Institute, Putra Infoport, Universiti Putra Malaysia, 43400, UPM Serdang, Selangor Darul Ehsan, Malaysia,

Cell No.: +60 12 654 5411

**Fig. S1** The radar diagram shows the reduction percentage (%) of the inhibitory effect (ZOI, mm) by neutralized CFS (n-CFS) produced by four LAB strains compared to raw CFS (r-CFS) against indicator strains. LP, L. plantarum NBRC 3070; LA, L. acidophilus ATCC 4356; LC, L. casei ATCC 393; LGG, L. rhamnosus GG ATCC 53103.

Figure 8: Reduction (%) of effectiveness of CFS produced by five LAB strains with antibacterial activity (ZOI, mm) when incubated for 16 h than for 24 h against seven indicator bacteria
