# Supplementary material for: Evaluation of Probiotic Properties and Antimicrobial Substances Produced by Five Lactic Acid Bacteria Against Foodborne and Spoilage Pathogens
Source: Microbiologyopen. 2026 Sep 25;15(5):e70052. doi: 10.1002/mbo3.70052 (PMC13613172; doi:10.1002/mbo3.70052)
Supplement: Supplementary file 2 [file MBO3-15-e70052-s002.docx]

**Evaluation of probiotic properties and antimicrobial substances produced by five lactic acid bacteria against foodborne and spoilage pathogens**

**MicrobiologyOpen**

Md. Moklesur Rahman^1,6^, Awis Qurni Sazili^1,2^, Siti Aqlima Ahmad^3^, Khalilah Abdul Khalil^4^, Mohammad Rashedi Ismail-Fitry^1,5^, and Md. Sazedul Karim Sarker^6^

^1^Halal Products Development, Halal Product Research Institute, Universiti Putra Malaysia, 43400, UPM Serdang, Selangor Darul Ehsan, Malaysia; ^2^Faculty of Agriculture, Universiti Putra Malaysia, 43400, UPM Serdang, Selangor Darul Ehsan, Malaysia; ^3^Faculty of Biotechnology and Bimolecular Science, Universiti Putra Malaysia, 43400, UPM Serdang, Selangor Darul Ehsan, Malaysia; ^4^Faculty of Applied Science, Universiti Teknologi MARA, 40450 Shah Alam, Selangor, Darul Ehsan, Malaysia; ^5^Faculty of Food Science and Technology, Universiti Putra Malaysia, 43400, UPM Serdang, Selangor Darul Ehsan, Malaysia; ^6^Bangladesh Livestock Research Institute (BLRI), Savar, Dhaka, Bangladesh.

**Corresponding author:** Awis Qurni Sazili (awis@upm.edu.my). Mailing address: Director, Halal Product Research Institute, Putra Infoport, Universiti Putra Malaysia, 43400, UPM Serdang, Selangor Darul Ehsan, Malaysia, Cell No.: +60 12 654 5411

**Table S9:** The comparative antimicrobial activity of r-CFS and BLIS (n-CFS) of four LAB strains against four pathogenic bacteria.

| LAB strains | *E. coli* | | p-value | *S. Typhimurium* | | P-value | *S. aureus* | | P-value | *P. aeruginosa* | | P-value | Pathogens  (r-CFS) | Pathogens  (n-CFS) |
| --- | --- | --- | --- | --- | --- | --- | --- | --- | --- | --- | --- | --- | --- | --- |
|  | r-CFS | n-CFS |  | r-CFS | n-CFS |  | r-CFS | n-CFS |  | r-CFS | n-CFS |  | P-value | |
| LP | 23.39±0.54^aA^ | 14.33±0.52^aX^ | 0.000 | 22.52±0.68^aAB^ | 15.33±0.51^aX^ | 0.001 | 20.34±0.55^aBC^ | 13.39±0.49^aX^ | 0.001 | 18.58±0.51^aC^ | 14.14±0.33^aX^ | 0.002 | 0.001 | 0.098 |
| LA | 19.69±0.76^bA^ | 13.57±0.50^aY^ | 0.003 | 17.77±0.67^bA^ | 15.55±0.36^aX^ | 0.043 | 19.65±0.58^aA^ | 15.46±0.45^aX^ | 0.005 | 17.61±0.51^aA^ | 14.25±0.44^aX^ | 0.008 | 0.083 | 0.038 |
| LC | 23.29±0.53^aA^ | 10.74±0.53^bY^ | 0.000 | 18.39±0.58^bB^ | 12.44±0.48^bXY^ | 0.001 | 20.55±0.49^aB^ | 14.58±0.70^aX^ | 0.002 | 18.60±0.42^aB^ | nd | - | 0.000 | 0.009 |
| LGG | 21.86±0.57^abA^ | 12.43±0.52^abX^ | 0.001 | 17.89±0.64^bBC^ | 14.30±0.47^abX^ | 0.011 | 20.40±0.56^aAB^ | 13.30±0.53^aX^ | 0.001 | 17.61±0.46^aC^ | 13.06±0.46^aX^ | 0.002 | 0.002 | 0.230 |
| P-value | 0.009 | 0.014 |  | 0.002 | 0.007 |  | 0.670 | 0.069 |  | 0.306 | 0.162 |  |  |  |

LP, *L. plantarum* NBRC 3070; LA, *L. acidophilus* ATCC 4356; LC, *L. casei* ATCC 393; LGG, *L. rhamnosus* GG ATCC 53103; r-CFS, raw liquid cell-free supernatant; n-CFS, neutralized cell-free supernatant; Lowercase letters (a and b) represent statistically significant differences of r-CFS and n-CFS among different LAB strains within each pathogenic bacteria strain. Uppercase letters (A, B, and C) represent statistically significant differences in r-CFS among pathogenic bacteria strains within each LAB strain. Uppercase letters (X and Y) represent statistically significant differences in n-CFS among different pathogenic bacteria strains within each LAB strain (n = 3, p < 0.05, Duncan’s test). SEM, the standard error of the mean.
