# Supplementary material for: Evaluation of Probiotic Properties and Antimicrobial Substances Produced by Five Lactic Acid Bacteria Against Foodborne and Spoilage Pathogens
Source: Microbiologyopen. 2026 Sep 25;15(5):e70052. doi: 10.1002/mbo3.70052 (PMC13613172; doi:10.1002/mbo3.70052)
Supplement: Supplementary file 3 [file MBO3-15-e70052-s003.docx]

**Evaluation of probiotic properties and antimicrobial substances produced by five lactic acid bacteria against foodborne and spoilage pathogens**

**MicrobiologyOpen**

Md. Moklesur Rahman^1,6^, Awis Qurni Sazili^1,2^, Siti Aqlima Ahmad^3^, Khalilah Abdul Khalil^4^, Mohammad Rashedi Ismail-Fitry^1,5^, and Md. Sazedul Karim Sarker^6^

^1^Halal Products Development, Halal Product Research Institute, Universiti Putra Malaysia, 43400, UPM Serdang, Selangor Darul Ehsan, Malaysia; ^2^Faculty of Agriculture, Universiti Putra Malaysia, 43400, UPM Serdang, Selangor Darul Ehsan, Malaysia; ^3^Faculty of Biotechnology and Bimolecular Science, Universiti Putra Malaysia, 43400, UPM Serdang, Selangor Darul Ehsan, Malaysia; ^4^Faculty of Applied Science, Universiti Teknologi MARA, 40450 Shah Alam, Selangor, Darul Ehsan, Malaysia; ^5^Faculty of Food Science and Technology, Universiti Putra Malaysia, 43400, UPM Serdang, Selangor Darul Ehsan, Malaysia; ^6^Bangladesh Livestock Research Institute (BLRI), Savar, Dhaka, Bangladesh.

**Corresponding author:** Awis Qurni Sazili (awis@upm.edu.my). Mailing address: Director, Halal Product Research Institute, Putra Infoport, Universiti Putra Malaysia, 43400, UPM Serdang, Selangor Darul Ehsan, Malaysia,

Cell No.: +60 12 654 5411

**Table S1** The growth and nature of LAB and pathogenic bacteria strains used for antibacterial activity test.

| Sl. No. | LAB and pathogenic bacteria strains | Accession no. | Growth condition | Gram’s nature |
| --- | --- | --- | --- | --- |
| LAB strains | | | | |
| 01 | *Lactiplantibacillus plantarum* | NBRC 3070 | MRS broth, 24 h at 37ºC | Gram-positive |
| 02 | *Lactobacillus acidophilus* | ATCC 4356 | MRS broth, 24 h at 37ºC |  |
| 03 | *Lacticaseibacillus casei* | ATCC 393 | MRS broth, 24 h at 37ºC |  |
| 04 | *Lacticaseibacillus rhamnosus* GG | ATCC 53103 | MRS broth, 24 h at 37ºC |  |
| 05 | *Bifidobacterium animalis* subsp. *lactis* | ATCC 27673 | MRS broth, 24 h at 37ºC |  |
| Pathogenic bacterial strains | | | | |
| 01 | *Escherichia coli* | ATCC 25922 | LB broth, 24 h at 37ºC | Gram-negative |
| 02 | *Salmonella enterica serovar* Typhimurium | ATCC 14028 | LB broth, 24 h at 30ºC |  |
| 03 | *Shigella Sonnei* | ATCC 25931 | LB broth, 24 h at 37ºC |  |
| 04 | *Pseudomonas aeruginosa* | ATCC 10145 | LB broth, 24 h at 28ºC |  |
| 05 | *Serratia marcescens* | ATCC 14756 | LB broth, 24 h at 37ºC |  |
| 06 | *Staphylococcus aureus* | ATCC 43300 | LB broth, 24 h at 37ºC | Gram-positive |
| 07 | *Bacillus cereus* | ATCC 14579 | LB broth, 24 h at 28ºC |  |

NBRC, Biological Resource Center, National Institute of Technology and Evaluation; ATCC, American Type Culture Collection; MRS, de Man, Rogosa, and Sharpe and LB, Luria Bertani broth
