# Supplementary material for: Evaluation of Probiotic Properties and Antimicrobial Substances Produced by Five Lactic Acid Bacteria Against Foodborne and Spoilage Pathogens
Source: Microbiologyopen. 2026 Sep 25;15(5):e70052. doi: 10.1002/mbo3.70052 (PMC13613172; doi:10.1002/mbo3.70052)
Supplement: Supplementary file 4 [file MBO3-15-e70052-s004.docx]

**Evaluation of probiotic properties and antimicrobial substances produced by five lactic acid bacteria against foodborne and spoilage pathogens**

**MicrobiologyOpen**

Md. Moklesur Rahman^1,6^, Awis Qurni Sazili^1,2^, Siti Aqlima Ahmad^3^, Khalilah Abdul Khalil^4^, Mohammad Rashedi Ismail-Fitry^1,5^, and Md. Sazedul Karim Sarker^6^

^1^Halal Products Development, Halal Product Research Institute, Universiti Putra Malaysia, 43400, UPM Serdang, Selangor Darul Ehsan, Malaysia; ^2^Faculty of Agriculture, Universiti Putra Malaysia, 43400, UPM Serdang, Selangor Darul Ehsan, Malaysia; ^3^Faculty of Biotechnology and Bimolecular Science, Universiti Putra Malaysia, 43400, UPM Serdang, Selangor Darul Ehsan, Malaysia; ^4^Faculty of Applied Science, Universiti Teknologi MARA, 40450 Shah Alam, Selangor, Darul Ehsan, Malaysia; ^5^Faculty of Food Science and Technology, Universiti Putra Malaysia, 43400, UPM Serdang, Selangor Darul Ehsan, Malaysia; ^6^Bangladesh Livestock Research Institute (BLRI), Savar, Dhaka, Bangladesh.

**Corresponding author:** Awis Qurni Sazili (awis@upm.edu.my). Mailing address: Director, Halal Product Research Institute, Putra Infoport, Universiti Putra Malaysia, 43400, UPM Serdang, Selangor Darul Ehsan, Malaysia

Cell No.: +60 12 654 5411

| **Table S8** The inhibitory potential (%inhibition) against *S. aureus* of the bioactive substances produced from five LAB strains incubated for 48 h at 30 and 37 ^o^C | | | | | | | | |
| --- | --- | --- | --- | --- | --- | --- | --- | --- |
| Temperature (^o^C) | Time (h) | LAB strains (±SEM) | | | | | SEM | P-value |
|  |  | LP | LA | LC | LGG | BAL |  |  |
| 30 | 12 | 34.71±2.608^bcC^ | 32.40±1.408^bC^ | 52.37±0.862^aB^ | 53.83±2.343^aB^ | 41.59±0.901^abC^ | 1.779 | 0.000 |
|  | 24 | 85.97±1.459^aB^ | 85.62±1.709^aB^ | 89.36±0.901^aA^ | 87.50±0.849^aA^ | 87.65±0.423^aB^ | 1.163 | 0.236 |
|  | 36 | 91.08±0.973^aA^ | 91.25±0.537^aA^ | 91.15±0.932^aA^ | 90.71±0.394^aA^ | 91.03±0.527^aA^ | 0.712 | 0.746 |
|  | 48 | 90.62±0.475^aA^ | 91.16±0.893^aAB^ | 90.72±0.658^aA^ | 90.76±0.910^aA^ | 90.70±0.448^aA^ | 0.705 | 0.790 |
| SEM | | 1.589 | 1.223 | 0.845 | 1.341 | 0.606 |  |  |
| P-value | | 0.000 | 0.000 | 0.000 | 0.000 | 0.000 |  |  |
| 37 | 12 | 35.86±1.769^bB^ | 40.53±1.782^abB^ | 45.10±1.362^aB^ | 46.72±1.741^aB^ | 40.92±1.462^abB^ | 1.633 | 0.006 |
|  | 24 | 90.52±0.704^aA^ | 90.70±0.619^aA^ | 90.78±0.447^aA^ | 90.79±0.586^aA^ | 89.75±0.380^aA^ | 0.560 | 0.667 |
|  | 36 | 89.16±0.431^aA^ | 88.87±0.411^aA^ | 89.79±0.432^aA^ | 89.69±0.125^aA^ | 88.58±0.465^aA^ | 0.393 | 0.216 |
|  | 48 | 89.48±0.778^aA^ | 89.21±0.587^aA^ | 89.27±0.300^aA^ | 87.84±0.521^aA^ | 88.17±0.362^aA^ | 0.537 | 0.192 |
| SEM | | 1.051 | 1.009 | 0.763 | 0.957 | 0.811 |  |  |
| P-value | | 0.000 | 0.000 | 0.000 | 0.000 | 0.000 |  |  |

^a-c^ Means with different superscript letters within the same row differ significantly (P < 0.05) between treatments. ^A-C^ Means with different superscript letters within the same column differ significantly (P < 0.01) among the incubation periods. Means are presented as SEM (standard error of the mean). LP, *L. plantarum* NBRC 3070; LA, *L. acidophilus* ATCC 4356; LC, *L. casei* ATCC 393; LGG, *L. rhamnosus* GG ATCC 53103; and BAL, *B. animalis* subsp. *lactis* ATCC 27673
