# Supplementary material for: Evaluation of Probiotic Properties and Antimicrobial Substances Produced by Five Lactic Acid Bacteria Against Foodborne and Spoilage Pathogens
Source: Microbiologyopen. 2026 Sep 25;15(5):e70052. doi: 10.1002/mbo3.70052 (PMC13613172; doi:10.1002/mbo3.70052)
Supplement: Supplementary file 5 [file MBO3-15-e70052-s005.docx]

**Evaluation of probiotic properties and antimicrobial substances produced by five lactic acid bacteria against foodborne and spoilage pathogens**

**MicrobiologyOpen**

Md. Moklesur Rahman^1,6^, Awis Qurni Sazili^1,2^, Siti Aqlima Ahmad^3^, Khalilah Abdul Khalil^4^, Mohammad Rashedi Ismail-Fitry^1,5^, and Md. Sazedul Karim Sarker^6^

^1^Halal Products Development, Halal Product Research Institute, Universiti Putra Malaysia, 43400, UPM Serdang, Selangor Darul Ehsan, Malaysia; ^2^Faculty of Agriculture, Universiti Putra Malaysia, 43400, UPM Serdang, Selangor Darul Ehsan, Malaysia; ^3^Faculty of Biotechnology and Bimolecular Science, Universiti Putra Malaysia, 43400, UPM Serdang, Selangor Darul Ehsan, Malaysia; ^4^Faculty of Applied Science, Universiti Teknologi MARA, 40450 Shah Alam, Selangor, Darul Ehsan, Malaysia; ^5^Faculty of Food Science and Technology, Universiti Putra Malaysia, 43400, UPM Serdang, Selangor Darul Ehsan, Malaysia; ^6^Bangladesh Livestock Research Institute (BLRI), Savar, Dhaka, Bangladesh.

**Corresponding author:** Awis Qurni Sazili (awis@upm.edu.my). Mailing address: Director, Halal Product Research Institute, Putra Infoport, Universiti Putra Malaysia, 43400, UPM Serdang, Selangor Darul Ehsan, Malaysia

Cell No.: +60 12 654 5411

| **Table S6** The variation in cell growth (OD_600nm_ value) of five LAB strains incubated for 48 h at 30 and 37 ^o^C | | | | | | | | |
| --- | --- | --- | --- | --- | --- | --- | --- | --- |
| Temperature (^o^C) | Time (h) | LAB strains (±SEM) | | | | | SEM | P-value |
|  |  | LP | LA | LC | LGG | BAL |  |  |
| 30 | 12 | 0.45±0.039^aC^ | 0.43±0.023^aD^ | 0.40±0.049^bC^ | 0.39±0.018^bC^ | 0.44±0.023^aC^ | 0.033 | 0.046 |
|  | 24 | 0.88±0.055^aB^ | 0.79±0.019^aC^ | 1.01±0.029^aB^ | 0.96±0.060^aB^ | 0.97±0.056^aB^ | 0.047 | 0.055 |
|  | 36 | 1.49±0.021^aA^ | 1.32±0.062^abB^ | 1.29±0.080^abA^ | 1.20±0.015^bA^ | 1.22±0.058^bA^ | 0.053 | 0.022 |
|  | 48 | 1.66±0.051^aA^ | 1.54±0.012^abA^ | 1.32±0.068^cA^ | 1.37±0.037^bcA^ | 1.35±0.043^bcA^ | 0.046 | 0.002 |
| SEM | | 0.044 | 0.035 | 0.060 | 0.037 | 0.047 |  |  |
| P-value | | 0.000 | 0.000 | 0.000 | 0.000 | 0.000 |  |  |
| 37 | 12 | 0.46±0.020^bcC^ | 0.54±0.020^bC^ | 0.38±0.015^cB^ | 0.35±0.015^cC^ | 0.65±0.023^aC^ | 0.019 | 0.000 |
|  | 24 | 1.32±0.042^aB^ | 1.22±0.019^aB^ | 1.16±0.036^aA^ | 1.17±0.050^aB^ | 1.18±0.052^aB^ | 0.041 | 0.106 |
|  | 36 | 1.54±0.043^aA^ | 1.46±0.044^aA^ | 1.32±0.055^aA^ | 1.37±0.046^aA^ | 1.36±0.053^aAB^ | 0.048 | 0.059 |
|  | 48 | 1.53±0.058^aA^ | 1.35±0.038^aAB^ | 1.38±0.092^aA^ | 1.48±0.055^aA^ | 1.45±0.053^aA^ | 0.062 | 0.288 |
| SEM | | 0.043 | 0.032 | 0.057 | 0.044 | 0.047 |  |  |
| P-value | | 0.000 | 0.000 | 0.000 | 0.000 | 0.000 |  |  |

^abc^ Means with different superscript letters within the same row differ significantly (P < 0.05) between treatments. ^A-D^ Means with different superscript letters within the same column differ significantly (P < 0.01) among the incubation periods. Means are presented as SEM (standard error of the mean). LP, *L. plantarum* NBRC 3070; LA, *L. acidophilus* ATCC 4356; LC, *L. casei* ATCC 393; LGG, *L. rhamnosus* GG ATCC 53103; and BAL, *B. animalis* subsp. *lactis* ATCC 27673
