# Supplementary material for: Evaluation of Probiotic Properties and Antimicrobial Substances Produced by Five Lactic Acid Bacteria Against Foodborne and Spoilage Pathogens
Source: Microbiologyopen. 2026 Sep 25;15(5):e70052. doi: 10.1002/mbo3.70052 (PMC13613172; doi:10.1002/mbo3.70052)
Supplement: Supplementary file 6 [file MBO3-15-e70052-s006.docx]

**Evaluation of probiotic properties and antimicrobial substances produced by five lactic acid bacteria against foodborne and spoilage pathogens**

**MicrobiologyOpen**

Md. Moklesur Rahman^1,6^, Awis Qurni Sazili^1,2^, Siti Aqlima Ahmad^3^, Khalilah Abdul Khalil^4^, Mohammad Rashedi Ismail-Fitry^1,5^, and Md. Sazedul Karim Sarker^6^

^1^Halal Products Development, Halal Product Research Institute, Universiti Putra Malaysia, 43400, UPM Serdang, Selangor Darul Ehsan, Malaysia; ^2^Faculty of Agriculture, Universiti Putra Malaysia, 43400, UPM Serdang, Selangor Darul Ehsan, Malaysia; ^3^Faculty of Biotechnology and Bimolecular Science, Universiti Putra Malaysia, 43400, UPM Serdang, Selangor Darul Ehsan, Malaysia; ^4^Faculty of Applied Science, Universiti Teknologi MARA, 40450 Shah Alam, Selangor, Darul Ehsan, Malaysia; ^5^Faculty of Food Science and Technology, Universiti Putra Malaysia, 43400, UPM Serdang, Selangor Darul Ehsan, Malaysia; ^6^Bangladesh Livestock Research Institute (BLRI), Savar, Dhaka, Bangladesh.

**Corresponding author:** Awis Qurni Sazili (awis@upm.edu.my). Mailing address: Director, Halal Product Research Institute, Putra Infoport, Universiti Putra Malaysia, 43400, UPM Serdang, Selangor Darul Ehsan, Malaysia

Cell No.: +60 12 654 5411

**Table S3** Antibiotics used in the study, along with their class and mode of antibacterial action

| **SL No.** | **Antibiotic (Disc code** | **Standard concentration (µg/ml)** | **Antimicrobial Class** | **Activity** |
| --- | --- | --- | --- | --- |
| 01 | Streptomycin (S-10) | 10 | Aminoglycosides | Ribosomal protein synthesis inhibitor |
| 02 | Norfloxacin (NOR-10) | 10 | Quinolones | Nucleic acid synthesis inhibitors |
| 03 | Ciprofloxacin (CIP-5) | 5 | Quinolones | DNA replication inhibitor |
| 04 | Gentamycin (CN-10 | 10 | Aminoglycosides | Protein Synthesis Inhibitor |
| 05 | Azithromycin (AZM-30) | 30 | Macrolides | Ribosomal protein synthesis inhibitor |
| 06 | Penicillin (P-1) | 10 | β-Lactams | Cell wall synthesis inhibitors |
| 07 | Tetracycline (TE-30) | 30 | Tetracyclines | Protein Synthesis Inhibitor |
