# Supplementary material for: Evaluation of Probiotic Properties and Antimicrobial Substances Produced by Five Lactic Acid Bacteria Against Foodborne and Spoilage Pathogens
Source: Microbiologyopen. 2026 Sep 25;15(5):e70052. doi: 10.1002/mbo3.70052 (PMC13613172; doi:10.1002/mbo3.70052)
Supplement: Supplementary file 7 [file MBO3-15-e70052-s007.docx]

**Evaluation of probiotic properties and antimicrobial substances produced by five lactic acid bacteria against foodborne and spoilage pathogens**

**MicrobiologyOpen**

Md. Moklesur Rahman^1,6^, Awis Qurni Sazili^1,2^, Siti Aqlima Ahmad^3^, Khalilah Abdul Khalil^4^, Mohammad Rashedi Ismail-Fitry^1,5^, and Md. Sazedul Karim Sarker^6^

^1^Halal Products Development, Halal Product Research Institute, Universiti Putra Malaysia, 43400, UPM Serdang, Selangor Darul Ehsan, Malaysia; ^2^Faculty of Agriculture, Universiti Putra Malaysia, 43400, UPM Serdang, Selangor Darul Ehsan, Malaysia; ^3^Faculty of Biotechnology and Bimolecular Science, Universiti Putra Malaysia, 43400, UPM Serdang, Selangor Darul Ehsan, Malaysia; ^4^Faculty of Applied Science, Universiti Teknologi MARA, 40450 Shah Alam, Selangor, Darul Ehsan, Malaysia; ^5^Faculty of Food Science and Technology, Universiti Putra Malaysia, 43400, UPM Serdang, Selangor Darul Ehsan, Malaysia; ^6^Bangladesh Livestock Research Institute (BLRI), Savar, Dhaka, Bangladesh.

**Corresponding author:** Awis Qurni Sazili (awis@upm.edu.my). Mailing address: Director, Halal Product Research Institute, Putra Infoport, Universiti Putra Malaysia, 43400, UPM Serdang, Selangor Darul Ehsan, Malaysia

Cell No.: +60 12 654 5411

**Table S4** Diameter (mm) of inhibition zones with well diffusion assay generated by whole bacterial cell cultures of studied LAB strains against selected indicator strains for 24 h of incubation.

|  | *E. coli* | *S. Typhimurium* | *S. aureus* | *S. Sonnei* | *S. aeruginosa* | *S. marcescens* | *B. cereus* | SEM | P-value |
| --- | --- | --- | --- | --- | --- | --- | --- | --- | --- |
| *L. plantarum* NBRC 3070 | 23.06^a,A^ | 22.52^a,AB^ | 20.41^a,CD^ | 19.60^a,D^ | 19.12^ab,D^ | 21.26^a,BC^ | 22.34^a,Ab^ | 0.428 | 0.000 |
| *L. acidophilus* ATCC 4356 | 22.95^a,A^ | 21.47^b,AB^ | 19.58^a,C^ | 19.12^ab,C^ | 20.67^a,BC^ | 22.33^a,A^ | 19.66^bc,C^ | 0.559 | 0.001 |
| *L. casei* ATCC 393 | 21.90^b,A^ | 20.14^c,BC^ | 19.35^a,C^ | 17.18^bc,D^ | 19.67^a,BC^ | 19.67^b,BC^ | 20.53^b,B^ | 0.320 | 0.000 |
| *L. rhamnosus* GG ATCC 53103 | 21.15^b,A^ | 18.96^d,B^ | 19.80^a,B^ | 15.87^c,C^ | 16.33^c,C^ | 21.33^a,A^ | 18.94^cd,B^ | 0.432 | 0.000 |
| *B. animalis* Subsp. *lactis* ATCC 27673 | 19.76^c,A^ | 19.40^cd,AB^ | 19.94^a,A^ | 16.30^c,D^ | 17.33^bc,CD^ | 18.18^c,ABC^ | 17.71^d,BCD^ | 0.540 | 0.002 |
| SEM | 0.417 | 0.412 | 0.560 | 0.925 | 0.811 | 0.653 | 0.642 |  |  |
| P-value | 0.000 | 0.000 | 0.094 | 0.007 | 0.002 | 0.001 | 0.000 |  |  |

Lowercase letters (a, b, c, and d) represent statistically significant differences among different LAB strains within each pathogenic bacteria strain. Uppercase letters (A, B, C, D, and D) represent statistically significant differences among different pathogenic bacteria strains within each LAB strain (n = 3, p < 0.05, Duncan’s test). SEM, The Standard error of the mean.
