# Supplementary material for: Evaluation of Probiotic Properties and Antimicrobial Substances Produced by Five Lactic Acid Bacteria Against Foodborne and Spoilage Pathogens
Source: Microbiologyopen. 2026 Sep 25;15(5):e70052. doi: 10.1002/mbo3.70052 (PMC13613172; doi:10.1002/mbo3.70052)
Supplement: Supplementary file 8 [file MBO3-15-e70052-s008.docx]

**Evaluation of probiotic properties and antimicrobial substances produced by five lactic acid bacteria against foodborne and spoilage pathogens**

**MicrobiologyOpen**

Md. Moklesur Rahman^1,6^, Awis Qurni Sazili^1,2^, Siti Aqlima Ahmad^3^, Khalilah Abdul Khalil^4^, Mohammad Rashedi Ismail-Fitry^1,5^, and Md. Sazedul Karim Sarker^6^

^1^Halal Products Development, Halal Product Research Institute, Universiti Putra Malaysia, 43400, UPM Serdang, Selangor Darul Ehsan, Malaysia; ^2^Faculty of Agriculture, Universiti Putra Malaysia, 43400, UPM Serdang, Selangor Darul Ehsan, Malaysia; ^3^Faculty of Biotechnology and Bimolecular Science, Universiti Putra Malaysia, 43400, UPM Serdang, Selangor Darul Ehsan, Malaysia; ^4^Faculty of Applied Science, Universiti Teknologi MARA, 40450 Shah Alam, Selangor, Darul Ehsan, Malaysia; ^5^Faculty of Food Science and Technology, Universiti Putra Malaysia, 43400, UPM Serdang, Selangor Darul Ehsan, Malaysia; ^6^Bangladesh Livestock Research Institute (BLRI), Savar, Dhaka, Bangladesh.

**Corresponding author:** Awis Qurni Sazili (awis@upm.edu.my). Mailing address: Director, Halal Product Research Institute, Putra Infoport, Universiti Putra Malaysia, 43400, UPM Serdang, Selangor Darul Ehsan, Malaysia

Cell No.: +60 12 654 5411

**Table S2** The pH values of r-CFS and n-CFS from LAB strains were obtained 24 hours ago and used to screen antimicrobial activity.

| SL No. | LAB identity | pH | |
| --- | --- | --- | --- |
|  |  | r-CFS | n-CFS |
| 01 | *Lactiplantibacillus plantarum* NBRC 3070 | 3.78±0.138 | 6.52±0.010 |
| 02 | *Lactobacillus acidophilus* ATCC 4356 | 3.83±0.133 | 6.50±0.015 |
| 03 | *Lacticaseibacillus casei* ATCC 393 | 3.91±0.035 | 6.52±0.021 |
| 04 | *Lacticaseibacillus. rhamnosus* GG ATCC 53103 | 3.84±0.109 | 6.51±0.012 |
| 05 | *Bifidobacterium animalis* subsp. *lactis* ATCC 27673 | 3.86±0.084 | 6.52±0.015 |

LAB, Lactic acid bacteria; r-CFS, raw cell-free supernatant; n-CFS, neutralized cell-free supernatant. Values were mean ± SD (n=3)
