# Supplementary material for: Evaluation of Probiotic Properties and Antimicrobial Substances Produced by Five Lactic Acid Bacteria Against Foodborne and Spoilage Pathogens
Source: Microbiologyopen. 2026 Sep 25;15(5):e70052. doi: 10.1002/mbo3.70052 (PMC13613172; doi:10.1002/mbo3.70052)
Supplement: Supplementary file 9 [file MBO3-15-e70052-s009.docx]

**Evaluation of probiotic properties and antimicrobial substances produced by five lactic acid bacteria against foodborne and spoilage pathogens**

**MicrobiologyOpen**

Md. Moklesur Rahman^1,6^, Awis Qurni Sazili^1,2^, Siti Aqlima Ahmad^3^, Khalilah Abdul Khalil^4^, Mohammad Rashedi Ismail-Fitry^1,5^, and Md. Sazedul Karim Sarker^6^

^1^Halal Products Development, Halal Product Research Institute, Universiti Putra Malaysia, 43400, UPM Serdang, Selangor Darul Ehsan, Malaysia; ^2^Faculty of Agriculture, Universiti Putra Malaysia, 43400, UPM Serdang, Selangor Darul Ehsan, Malaysia; ^3^Faculty of Biotechnology and Bimolecular Science, Universiti Putra Malaysia, 43400, UPM Serdang, Selangor Darul Ehsan, Malaysia; ^4^Faculty of Applied Science, Universiti Teknologi MARA, 40450 Shah Alam, Selangor, Darul Ehsan, Malaysia; ^5^Faculty of Food Science and Technology, Universiti Putra Malaysia, 43400, UPM Serdang, Selangor Darul Ehsan, Malaysia; ^6^Bangladesh Livestock Research Institute (BLRI), Savar, Dhaka, Bangladesh.

**Corresponding author:** Awis Qurni Sazili (awis@upm.edu.my). Mailing address: Director, Halal Product Research Institute, Putra Infoport, Universiti Putra Malaysia, 43400, UPM Serdang, Selangor Darul Ehsan, Malaysia

Cell No.: +60 12 654 5411

| **Table S7** The change in pH value of five LAB strains incubated for 48 h at 30 and 37 ^o^C | | | | | | | | |
| --- | --- | --- | --- | --- | --- | --- | --- | --- |
| Temperature (^o^C) | Time (h) | LAB strains (±SEM) | | | | | SEM | P-value |
|  |  | LP | LA | LC | LGG | BAL |  |  |
| 30 | 12 | 5.41±0.132^aA^ | 5.37±0.072^aA^ | 4.90±0.098^bA^ | 5.00±0.084^abA^ | 4.99±0.049^abA^ | 0.091 | 0.005 |
|  | 24 | 4.89±0.050^abB^ | 4.92±0.026^aB^ | 4.15±0.098^cB^ | 4.41±0.133^cB^ | 4.49±0.101^bcB^ | 0.090 | 0.001 |
|  | 36 | 3.83±0.024^bC^ | 3.98±0.048^abC^ | 3.98±0.020^abB^ | 4.00±0.024^abC^ | 4.06±0.056^aC^ | 0.037 | 0.018 |
|  | 48 | 3.79±0.015^cC^ | 3.84±0.035^abC^ | 3.87±0.028^abB^ | 3.91±0.023^aC^ | 3.94±0.017^aC^ | 0.025 | 0.012 |
| SEM | | 0.072 | 0.049 | 0.071 | 0.081 | 0.063 |  |  |
| P-value | | 0.000 | 0.000 | 0.000 | 0.000 | 0.000 |  |  |
| 37 | 12 | 5.36±0.157^aA^ | 5.29±0.139^aA^ | 5.18±0.086^aA^ | 5.15±0.070^aA^ | 5.18±0.085^aA^ | 0.113 | 0.668 |
|  | 24 | 3.84±0.027^aB^ | 3.92±0.021^aB^ | 3.93±0.027^aB^ | 3.90±0.024^aB^ | 3.91±0.026^aB^ | 0.025 | 0.167 |
|  | 36 | 3.80±0.009^aB^ | 3.80±0.012^aB^ | 3.85±0.018^aB^ | 3.80±0.015^aB^ | 3.78±0.015^aB^ | 0.014 | 0.071 |
|  | 48 | 3.80±0.015^aB^ | 3.81±0.021^aB^ | 3.81±0.009^aB^ | 3.83±0.018^aB^ | 3.79±0.006^aCB^ | 0.015 | 0.542 |
| SEM | | 0.080 | 0.071 | 0.046 | 0.039 | 0.045 |  |  |
| P-value | | 0.000 | 0.000 | 0.000 | 0.000 | 0.000 |  |  |

^abc^ Means that different superscript letters within the same row differ significantly (P < 0.05) between treatments. ^ABC^ Means with different superscript letters within the same column differ significantly (P < 0.01) among the incubation periods. Means are presented as SEM (standard error of the mean). LP, *L. plantarum* NBRC 3070; LA, *L. acidophilus* ATCC 4356; LC, *L. casei* ATCC 393; LGG, *L. rhamnosus* GG ATCC 53103; and BAL, *B. animalis* subsp. *lactis* ATCC 27673
