# Supplementary material for: Evaluation of Probiotic Properties and Antimicrobial Substances Produced by Five Lactic Acid Bacteria Against Foodborne and Spoilage Pathogens
Source: Microbiologyopen. 2026 Sep 25;15(5):e70052. doi: 10.1002/mbo3.70052 (PMC13613172; doi:10.1002/mbo3.70052)
Supplement: Supplementary file 10 [file MBO3-15-e70052-s010.docx]

**Evaluation of probiotic properties and antimicrobial substances produced by five lactic acid bacteria against foodborne and spoilage pathogens**

**MicrobiologyOpen**

Md. Moklesur Rahman^1,6^, Awis Qurni Sazili^1,2^, Siti Aqlima Ahmad^3^, Khalilah Abdul Khalil^4^, Mohammad Rashedi Ismail-Fitry^1,5^, and Md. Sazedul Karim Sarker^6^

^1^Halal Products Development, Halal Product Research Institute, Universiti Putra Malaysia, 43400, UPM Serdang, Selangor Darul Ehsan, Malaysia; ^2^Faculty of Agriculture, Universiti Putra Malaysia, 43400, UPM Serdang, Selangor Darul Ehsan, Malaysia; ^3^Faculty of Biotechnology and Bimolecular Science, Universiti Putra Malaysia, 43400, UPM Serdang, Selangor Darul Ehsan, Malaysia; ^4^Faculty of Applied Science, Universiti Teknologi MARA, 40450 Shah Alam, Selangor, Darul Ehsan, Malaysia; ^5^Faculty of Food Science and Technology, Universiti Putra Malaysia, 43400, UPM Serdang, Selangor Darul Ehsan, Malaysia; ^6^Bangladesh Livestock Research Institute (BLRI), Savar, Dhaka, Bangladesh.

**Corresponding author:** Awis Qurni Sazili (awis@upm.edu.my). Mailing address: Director, Halal Product Research Institute, Putra Infoport, Universiti Putra Malaysia, 43400, UPM Serdang, Selangor Darul Ehsan, Malaysia

Cell No.: +60 12 654 5411

**Table S5** Co-aggregation (%) of lactic acid bacteria (LAB) strains with *E. coli* ATCC 25922 and *S. aureus* ATCC 43300 after incubating for 2 and 5 h at 37 °C.

| Lactic acid bacteria | Strains | Pathogens tested | | | |
| --- | --- | --- | --- | --- | --- |
|  |  | *E. coli* ATCC 25922 | | *S. aureus* ATCC 43300 | |
|  |  | 2 h | 5 h | 2 h | 5 h |
| *L. plantarum* | NBRC 3070 | 25.30±3.86^a^ | 27.63±2.46^xy^ | 24.87±4.41^ab^ | 29.88±1.88^yz^ |
| *L. acidophilus* | ATCC 4356 | 8.72±1.46b^c^ | 17.31±4.04^z^ | 21.87±0.64^bc^ | 24.19±2.08^z^ |
| *L. casei* | ATCC 393 | 3.55±0.56^c^ | 31.79±1.65^x^ | 15.82±5.06^bc^ | 38.30±3.92^x^ |
| *L. rhamnosus* GG | ATCC 53103 | 7.96±1.65b^c^ | 24.32±3.94^y^ | 13.18±1.76^c^ | 29.48±5.61^yz^ |
| *B. animalis* subsp. *lactis* | ATCC 27673 | 12.27±2.54^b^ | 31.80±5.55^x^ | 31.65±3.66^a^ | 36.66±4.48^xy^ |
| SEM | | 1.330 | 1.481 | 2.032 | 0.810 |
| P-value | | 0.000 | 0.004 | 0,001 | 0.007 |

The results shown are the means of three replications ± SD. Lowercase letters (a-c and x-z) represent statistically significant differences in pathogens tested between the LAB strain (n = 3, p < 0.05, Duncan’s test). SEM, the standard error of the mean.
